# Autoantibodies in long COVID in a black/mixed population compared with recovered and pre-pandemic controls

**DOI:** 10.3389/fimmu.2025.1684482

**Published:** 2025-12-11

**Authors:** Jessica de Jesus Silva, Laila Sampaio Horta, Sayonara Melo Viana, Ana Beatriz Cazé, Isabela S. Oliveira, Mariana M. Pereira, Natalie Antas Nascimento, Blenda de Jesus Pereira, Ícaro Bonyek-Silva, Sara Nunes de Oliveira Araújo, Ananda Isis Lima de Marinho, Juqueline Rocha Cristal, Vishal Rao, Camila Coelho, Thiago Cerqueira-Silva, Kelen Cristina Ribeiro Malmegrim, Aquiles Camelier, Cristina Ribeiro de Barros Cardoso, Natalia Machado Tavares, Manoel Barral-Netto, Aldina Barral, Cynara Barbosa, Viviane Sampaio Boaventura

**Affiliations:** 1 Laboratório de Medicina e Saúde Pública de Precisão, Fundação Oswaldo Cruz, Salvador, Brazil; 2 Universidade Federal da Bahia, Salvador, Brazil; 3 Microbiology Department, Icahn School of Medicine, Mount Sinai Hospital, New York City, NY, United States; 4 London School of Hygiene & Tropical Medicine, London, United Kingdom; 5 School of Pharmaceutical Sciences of Ribeirão Preto, University of São Paulo, Ribeirão Preto, Brazil; 6 Post-covid outpatient Clinic at Octavio Mangabeira Hospital, Salvador, Brazil

**Keywords:** autoantibodies, long Covid, COVID-19, anti-cardiolipin, biomarkers

## Abstract

**Introduction:**

Long COVID (LC), a clinical condition marked by persistent and new symptoms after infection with severe acute respiratory syndrome coronavirus 2 (SARS-CoV-2), affects up to 10-20% of infected individuals. Although autoimmunity has been proposed as a key mechanism, the specific role of circulating autoantibodies in LC remains unclear. We characterized the autoantibody profiles in individuals with LC and assessed their association with persistent post-COVID symptoms, in comparison to recovered patients and pre-pandemic healthy controls (PPHC).

**Methods:**

We analyzed 17 autoantibodies in a cohort of 220 pre-pandemic controls and 291 COVID-19 patients, targeting self-antigens. Of those, 237 patients presented symptoms for a month or more after the onset of SARS-CoV-2 infection (long COVID patients), and 54 individuals recovered from the initial infection without chronic symptoms. Autoantibody frequencies and associations with clinical variables were assessed using logistic regression and subgroup analyses.

**Results:**

Autoantibody prevalence was higher in recovered individuals (37%) than in LC patients (24%) or PPHC (19%). While certain autoantibodies such as a-cardiolipin (a-CL) IgM, a-AML IgG, a-SSA IgG and a-SSB IgG were elevated in some COVID-19 patients, they were not significantly different in LC. The most frequently detected autoantibody was a-CL IgM, found across all groups and especially in individuals that fully recovered from COVID-19. However, a-CL did not differentiate individuals with long COVID or correlate with symptom persistence but was associated with the occurrence of dysphagia and anorexia as symptoms. No correlation was observed between autoantibody presence and disease severity.

**Discussion:**

These findings do not support a primary pathogenic role for the evaluated autoantibodies in LC and emphasize the need for longitudinal studies to explore their temporal dynamics and interaction with other immunological or clinical factors involved in post-COVID-19 conditions.

## 1 Introduction

Five years into the COVID-19 pandemic, over 777 million cases have been reported worldwide. An estimated 10–20% of individuals develop long COVID (LC), a condition defined by the persistence or emergence of new symptoms lasting more than three months after the acute phase (1). Despite its widespread burden on individuals and health systems, the pathophysiological mechanisms underlying LC remain poorly understood (2).

Among the leading hypotheses, autoimmune dysregulation has emerged as a compelling contributor to LC. SARS-CoV-2-induced inflammation may disrupt immune tolerance, potentially leading to cross-reactive responses against self-antigens via mechanisms such as molecular mimicry (3, 4). Several studies have documented the presence of autoantibodies during the acute phase of COVID-19, which are associated with the severity of the infection (5–7).

The immunogenicity of COVID-19 associated autoantibodies lies in their capacity to recognize self-antigens and drive tissue damage. For instance, a-DNA antibodies, classically linked to systemic lupus erythematosus (SLE), may emerge through NETosis or molecular mimicry, leading to immune complex formation, complement activation, and organ inflammation (8). Similarly, a-Scl-70 antibodies, directed against topoisomerase I and typically associated with systemic sclerosis, may be induced by antigen exposure following SARS-CoV-2-related pulmonary injury, potentially contributing to post-COVID-19 fibrosis (9).

Autoantibodies such as a-MPO, a hallmark of ANCA-associated vasculitis, have also been reported in severe COVID-19, likely resulting from neutrophil activation, cytokine storms, and NET formation (8). Antiphospholipid antibodies, including a-CL and a-β2GP1 have gained attention due to their association with thrombotic complications and antiphospholipid syndrome (APS). In particular, a-CL IgM, known for its immunogenicity, may be triggered by polyclonal activation or mimicry mechanisms during persistent infections such as HIV, syphilis, and now possibly SARS-CoV-2 (10–12).

Other autoantibodies have been reported in the post-COVID-19 context. Anti-smooth muscle antibody (a-SM), associated with autoimmune hepatitis, may arise from liver injury induced by SARS-CoV-2. Similarly, anti-ribonucleoprotein (RNP) antibodies, frequent in SLE, could be induced by viral components that resemble nuclear autoantigens (2). These findings support a model in which SARS-CoV-2 infection precipitates or amplifies autoreactivity, potentially contributing to the pathogenesis of LC and related rheumatologic conditions.

Despite these insights, the profile of circulating autoantibodies in LC remains poorly understood. Complicating interpretation is the fact that autoantibodies can be found in healthy individuals, often without clinical autoimmune manifestations (13). Moreover, few studies included well-matched control groups of pre-pandemic individuals and those who recovered from COVID-19 without sequelae, limiting the ability to distinguish LC specific signatures from general post-viral immunity.

Here, we investigate the repertoire of circulating autoantibodies in individuals with LC, comparing them to both recovered individuals and pre-pandemic controls, with the aim of uncovering potential immunological signatures associated with persistent post-COVID-19 symptoms. Clarifying the role of autoantibodies in LC is crucial for advancing diagnostic biomarkers and therapeutic strategies.

## 2 Methods

### 2.1 Study population

The study included 291 serum and plasma samples from participants recruited at the Post-COVID Center in Salvador, Bahia (Cohort 1). Serum and plasma samples were collected in the post-acute phase of infection between August 2020 and March 2022, with a median of 3 months after the onset of acute COVID-19 symptoms (range: 1–10 months). A pre-pandemic healthy control group (PPHC; Cohort 2) comprised 220 serum samples obtained from a 2011 serosurvey conducted in Jiquiriça, Bahia.

**Figure 1:**
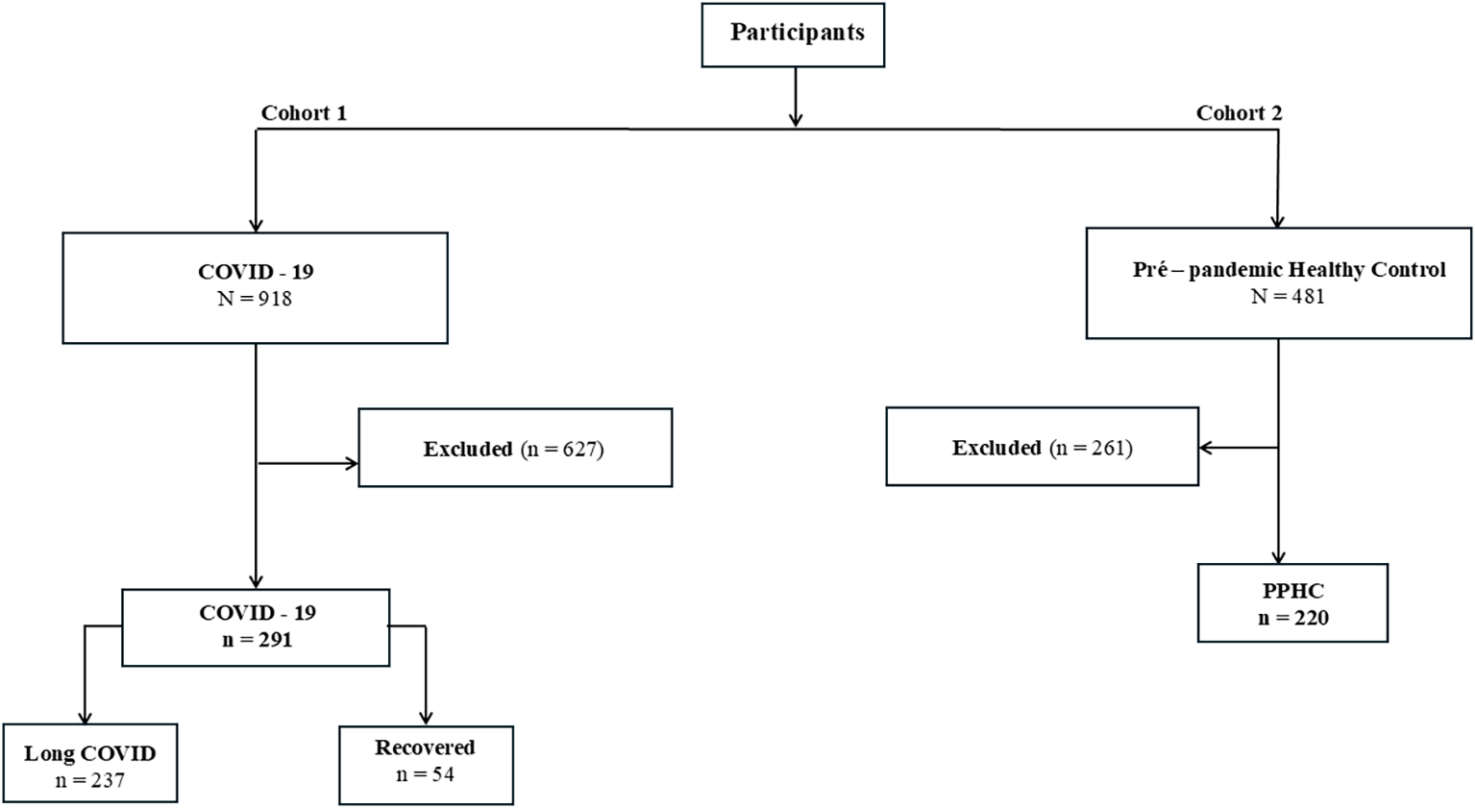
Flowchart of participant selection included in the study. Cohort 1 included individuals with a history of COVID-19 infection (n = 918); 291 were selected after applying exclusion criteria and classified into two groups: long COVID (n = 237) and Recovered (n = 54). Cohort 2 comprised pre-pandemic healthy individuals (n = 481), with 220 participants included. Participants from cohort 1 were matched by age and sex.

Participants were excluded from the analysis based on predefined eligibility criteria and data availability. In Cohort 1 (COVID-19), individuals without laboratory-confirmed SARS-CoV-2 infection (n = 10), without available serum or plasma samples (n = 27), not meeting matching criteria (n = 380), or with missing data (n = 210) were excluded, totaling 627 exclusions. In Cohort 2 (PPHC), participants without available biological samples (n = 25) or with incomplete sociodemographic data (n = 236) were excluded, resulting in 261 exclusions (**Figure 1**).

Participants had one of the following positive diagnostic tests for COVID-19: 1) Reverse Transcription followed by Polymerase Chain Reaction (RT-PCR); 2) IgM/IgG serology; 3) Rapid Antigen Test (RATs); and 4) Computed Tomography (CT) and suggestive clinical manifestations. There were 237 participants with long COVID (LC), who had persistent symptoms for more than a month, and 54 participants who recovered from COVID-19. The participants analyzed presented mild, moderate, or severe symptoms during the acute phase of COVID-19. To reduce potential biases related to demographic factors, LC and recovered participants were matched by sex and age. The pre-pandemic participants belonged to a different cohort and were not matched to the LC and recovered groups.

The study design and conduct complied with all relevant regulations (Research Ethics Committee of the Gonçalo Moniz Research Center - IGM/FIOCRUZ/BA) regarding the use of human study participants and was conducted in accordance with the criteria set by the Declaration of Helsinki. We obtained written informed consent from all patients. The project was approved by the Research Ethics Committee of the Fiocruz Gonçalo Moniz Institute - IGM/FIOCRUZ/BA in October 2020 (no. 4.315.319/2020) and December 2020 (no. 4.442.110/2020), respectively.

### 2.2 Sociodemographic and clinical data

Data were collected using structured questionnaires in Research Electronic Data Capture (REDCap). This included socio demographic information (gender, age, ethnicity, body mass index) and clinical details (COVID-19 symptoms and severity, comorbidities, current medications, and COVID-19 vaccination history). The severity of COVID-19 cases during the acute phase was classified as follows: 1) mild cases (participants not requiring hospitalization), 2) moderate cases (participants admitted to the ward), and 3) severe cases (participants admitted to the ICU). This classification was based on clinical support criteria, aligned with the Centers for Disease Control (CDC) guidelines (14). Individuals that presented symptoms for a month or more were considered as LC patients, using CDC guidelines from 2020. We also show data from LC patients defined as presenting symptoms for three months or more, according to CDC guidelines from 2024, on **Supplementary Tables S1**-**S3**, but we did not observe a significant difference from both stratifications.

### 2.3 Autoantibody analysis

Serum or plasma samples from participants were analyzed to evaluate the expression of 17 autoantibodies classes (a-β2GP1 IgG, a-β2GP1 IgM, a-CL IgG, a-CL IgM, a-SM IgG, a-SCL-70 IgG, a-SSA IgG, a-SSB IgG, a-MPO IgG, a-RNP IgG, a-dsDNA IgG, a-AML IgG, a-MT IgG, a-ANCA IgG, a-CP IgG, a-FAN IgG, a-LKM IgG). Autoantibody quantification was conducted at the Federal University of Bahia and at the Gonçalo Moniz Institute - Fiocruz-BA.

ELISA tests included anti-β2-glycoprotein I IgG and IgM (a-β2GP1 IgG, a-β2GP1 IgM; CAT #ORG521), anti-cardiolipin IgG and IgM (a-CL IgG, a-CL IgM; CAT #215G/M), anti-myeloperoxidase IgG (a-MPO IgG; CAT #ORG519), anti-SSA/Ro IgG (a-SSA IgG; CAT #ORG508), anti-SSB/La IgG (a-SSB IgG; CAT #ORG509), anti-ribonucleoprotein (a-RNP IgG (a-RNP IgG; CAT #ORG510), anti-Sm IgG (a-SM IgG; CAT #ORG510), anti-topoisomerase I IgG (a-SCL-70 IgG; CAT #ORG212), and anti-double-stranded DNA IgG (a-DNA IgG; CAT #ORG604), using commercial kits from ORGENTEC Diagnostika GmbH (Mainz, Germany). For a-β2GP1 IgG/IgM and a-CL IgG/IgM, the cut-off for positivity was set at ≥20 IU/mL, in accordance with the standard criteria for the diagnosis of antiphospholipid syndrome (APS) (15, 16). For the other autoantibodies, cut-offs were applied according to the manufacturer’s recommendations: ≥10 IU/mL for a-MPO, and ≥25 IU/mL for a-SSA, a-SSB, a-SM, a-SCL-70, and a-RNP70. ELISA assays were performed by allowing the autoantibodies to bind to solid-phase antigens, followed by washing steps and addition of enzyme substrate for quantification through color change or measurable signal.

Indirect immunofluorescence (IIF) assays for the detection of anti-mitochondrial (a-AMT), anti-smooth muscle (a-AML), anti-parietal cell (a-CP), anti-liver-kidney microsomal antibody (a-LKM), and antinuclear antibodies (a-FAN) were performed using commercial kits from BioSystems S.A. CAT#44648 (Curitiba, Brazil). For immunofluorescence assays, positivity was based on the presence or absence of fluorescent signal for the analyzed autoantibody. Samples were incubated to allow autoantibodies to bind to the substrate, followed by washing and incubation with fluorophore-conjugated secondary antibodies.

### 2.4 Statistical analysis

Categorical variables were presented as absolute and relative frequencies. The normality of continuous variables was determined using histograms and the Shapiro-Wilk test. Differences in proportions between groups were evaluated using Pearson’s chi-square test, and when any expected cell count was <5, Fisher’s exact test was applied. A significance level of *p* < 0.05 was considered. For binary logistic regression, clinical manifestations were grouped into clusters: 1) cardiorespiratory (dyspnea, expectoration, dysphonia, cough, and chest pain); 2) systemic involvement (fatigue, body pain, hair loss, dizziness, and motor limitation); 3) neurological (headache, insomnia, memory loss, anosmia, and ageusia); and 4) Dysphagia/Anorexia. Analyses were performed in R Studio (version 4.3.2) and GraphPad Prism (version 8.0.2).

## 3 Results

### 3.1 Sociodemographic data

The majority of participants in both the LC and recovered groups self-identified as having Mixed or Black ancestry (87.8% and 81.5%, respectively), while smaller proportions reported White, Asian, or Indigenous ancestry. Nearly half of those identifying as Black or Mixed ancestry experienced severe COVID-19. Among these individuals, approximately 25% exhibited detectable autoantibodies. Due to the limited number of participants with other ancestries, comparisons of autoantibody expression across different ethnic groups were not performed.

In the PPHC group, 60% identified as mixed or Black ancestry, 19.5% as White, and 20.5% did not report race/ethnicity. LC and recovered groups were demographically matched with regard to age and self-identified race/ethnicity (**Table 1**).

**Table 1:** Demographic and clinical characteristics of participants with long COVID, recovered individuals, and pre-pandemic healthy controls.

Characteristic	COVID-19		
	LC	Recovered	PPHC
	N = 237	N = 54	N = 220
Women (%)	104 (43.9%)	21 (38.9%)	112 (50.9%)
Age (median, IQR)	54 (43, 64)	53 (40, 63)	40 (24, 54)
Race			
Mixed	132 (55.7%)	23 (42.6%)	75 (34.1%)
Black	76 (32.1%)	21 (38.9%)	57 (25.9%)
White	25 (10.5%)	8 (15%)	43 (19.5%)
Asian	4 (1.7%)	1 (1.9%)	-
Indigenous	-	1 (1.9%)	
Not declared	-	-	45 (20.5%)
COVID-19 vaccine			
Yes	33 (13.9%)	14 (25.9%)	-
No	204 (86.1%)	40 (74.1%)	-
BMI	28.8 (25.7, 32.8)	28.6 (24.7, 31.5)	-
Months after disease onset (median, IQR)	2.7 (1.8, 4.1)	3.0 (1.6, 4.4)	-
Severity levels			
Mild	57 (24.1%)	19 (35.2%)	-
Moderate	68 (28.7%)	19 (35.2%)	-
Severe	112 (47.3%)	16 (29.6%)	-
COVID-19 tests			
RT-PCR	204 (86.1%)	50 (92.6%)	-
Sorology IgM/IgG	6 (2.5%)	0 (0%)	-
Rapid Antigen Test	22 (9.3%)	3 (5.6%)	-
Clinical-epidemiologic	5 (2.1%)	1 (1.9%)	-
Comorbidities			-
Hypertension	108 (45.6%)	26 (48.1%)	-
DM	53 (22.4%)	13 (24.1%)	-
Cardiopathy	20 (8.4%)	4 (7.4%)	-
Psychiatric disorder	26 (11%)	4 (7.4%)	-
Asthma	13 (5.5%)	0 (0%)	-

Data are shown as n (%) or median (Q1–Q3). “Not declared” indicates missing race/ethnicity information. “Vaccination” refers to at least one COVID-19 vaccine dose before sample collection. Abbreviations: LC, long COVID; PPHC, pre-pandemic healthy controls; IQR, interquartile range; BMI, body mass index; RT-PCR, reverse transcription polymerase chain reaction; IgM/IgG, immunoglobulin M/G; DM, diabetes mellitus.

Median age was comparable between the LC (54 years, IQR 43–64) and recovered (53 years, IQR 40–63) groups, whereas the PPHC group was younger (median 40 years, IQR 24–54). Female participants comprised 43.9% of the LC group, 38.9% of the recovered group, and 50.9% of the PPHC group. At the time of sample collection, only 33 individuals in the LC group (14%) and 14 in the recovered group (26%) had received one dose or more of a COVID-19 vaccine. This reflects the fact that participant recruitment occurred prior to the widespread availability of COVID-19 vaccines. The limited exposure to vaccination represents an advantage for our study, as it minimizes a potential confounding factor in the analysis of immune responses following natural infection.

During the acute phase of COVID-19, a higher proportion of individuals who later developed LC experienced severe illness compared to those who recovered without persistent symptoms (47.3% vs. 29.6%). Despite this difference in disease severity, the prevalence of comorbid conditions was comparable between the LC and recovered groups. Hypertension was observed in 45.6% of LC participants and 48.1% of recovered individuals; diabetes mellitus in 22.4% and 24.1%, respectively; cardiopathy in 8.4% and 7.4%; psychiatric disorders in 11% and 7.4%; and asthma in 5.5% and 0% (**Table 1**). Notably, among participants with diabetes, insulin use was more frequent in the LC group (28%, 15/53) compared to the recovered group (7.6%, 1/13; data not shown) (**Table 1**). One patient experienced a venous thromboembolism event during follow-up. The patient was hospitalized during the acute phase of COVID-19, and the thromboembolism event occurred one month after disease onset, suggesting it was linked to a post-COVID-19 complication.

The most frequent clinical manifestations reported by patients with LC were fatigue and myalgia. Other commonly described symptoms included memory impairment, headache, sleep disturbances, and dyspnea. Neurological, psychiatric, and gastrointestinal symptoms were also observed, though less frequently. Additionally, a few patients experienced venous thromboembolism events during follow-up (**Supplementary Figure S4**).

### 3.2 Frequency of autoantibodies and classification of COVID-19 outcome

**Figure 2:**
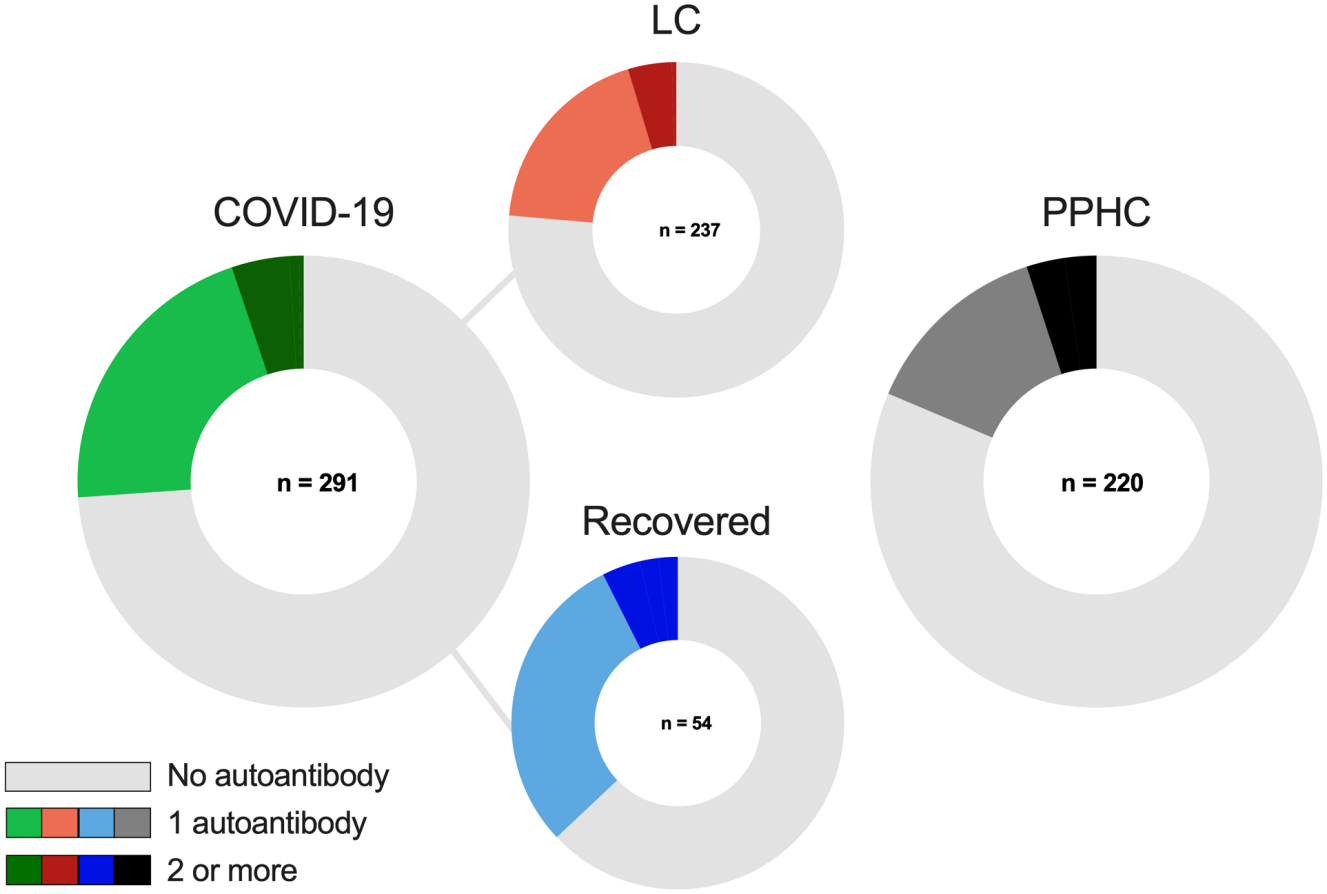
Distribution of individuals with 0, 1, or ≥2 autoantibodies in the total COVID-19 (n = 291), LC (n = 237), PPHC (n = 220), and recovered (n = 54) groups. Among participants with COVID-19, 21% had one autoantibody and 5% had two or more. In the LC, recovered, and PPHC groups, the proportions with one autoantibody were 19%, 30%, and 14%, and those with two or more were 5%, 7%, and 5%, respectively.

Autoantibody positivity rates were higher in the overall COVID-19 group (26.1% – 76/291) than in the PPHC (18.6% – 41/220; *p =* 0.046) (**Figure 2**).When analyzing subgroups, 23.6% (56/237) of individuals with long COVID tested positive for at least one autoantibody, while the proportion was higher among those who had recovered from acute infection, with 37% (20/54) testing positive. In addition to exhibiting a higher overall frequency of autoantibody production compared to PPHC, a greater proportion of recovered individuals produced multiple autoantibodies, indicating a broader autoreactive immune profile associated with SARS-CoV-2 exposure, independent of persistent symptoms (**Figure 2**).

**Figure 3:**
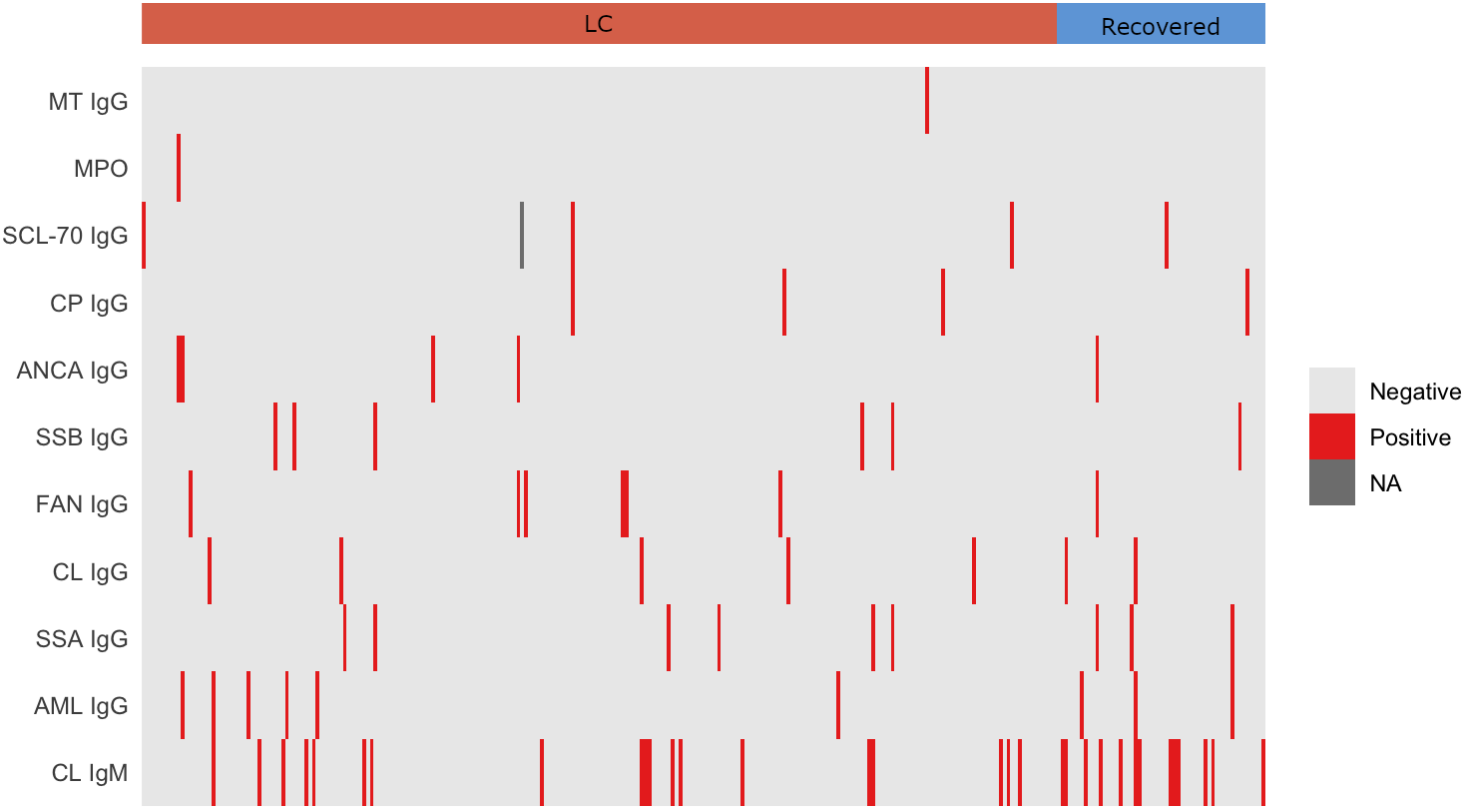
Overview of autoantibodies in individuals with LC and Recovered from COVID-19. The top 11 autoreactive antibodies are displayed in ascending order of positivity. Each column represents an individual with long COVID or a recovered participant. Cell colors indicate antibody titers below (light grey) or above (red) the predefined cutoff for each antibody, while missing data are shown in grey. NA = not available.

Among the 17 autoantibodies analyzed, the most prevalent across all groups were a-CL (IgG and IgM), a-AML IgG, a-SSA IgG, a-SSB IgG, a-β2GP1 IgM, a-FAN IgG and a-dsDNA IgG. Notably, a-CL IgM was the most frequently detected autoantibody overall, showing a significantly higher prevalence in recovered individuals (24.1%) than in those with LC (8.0%) or PPHC (8.1%; *p* = 0.001; **Supplementary Table S5**). When LC and recovered participants were stratified by the severity of their acute disease, 31.2% of recovered individuals who had experienced severe COVID-19 were a-CL IgM–positive, compared with 9.8% of those with LC (**Table 2**). In contrast, a-β_2_GP1 IgG was detected exclusively in the PPHC group (3.2%), whereas a-β_2_GP1 IgM was more common in PPHC (7.3%) than among individuals who had COVID-19 (1.0%). Levels of SSA-IgG and AML-IgG were higher in recovered participants (5.5%) compared with LC (2.5%) and PPHC (0.5%) groups. Other autoantibodies occurred at low frequencies across all groups and did not differ significantly. Overall, no consistent pattern in autoantibody distribution was observed among LC, recovered, and pre-pandemic participants (**Figure 3**; **Supplementary Table S5**), suggesting that LC was not associated with elevated autoantibody titers.

**Table 2:** Frequency of autoantibodies in COVID-19 participants. Long COVID patients included here exhibited symptoms for one month or more after SARS-CoV-2 infection onset.

Autoantibodies	COVID - 19					
	Long COVID			Recovered		
	Mild/Moderate	Severe	P-value¹	Mild/Moderate	Severe	P-value²
	N = 125	N = 112		N = 38	N = 16	
β2GP1 IgG	0 (0%)	0 (0%)	**-**	0 (0%)	0 (0%)	**-**
β2GP1 IgM	0 (0%)	2 (1.7%)	0.2223	1 (2.6%)	0 (0%)	>0.9999
CL IgG	3 (2.4%)	2 (1.7%)	>0.9999	2 (5.6%)	0 (0%)	>0.9999
CL IgM	8 (6.4%)	11 (9.8%)	0.3329	8 (21%)	5 (31.2%)	0.4235
SM IgG	0 (0%)	0 (0%)	**-**	0 (0%)	0 (0%)	**-**
SCL-70 IgG	1 (0.8%)	2 (1.8%)	0.6053	0 (0%)	0 (0%)	**-**
SSA IgG	3 (2.4%)	3 (2.7%)	>0.9999	2 (5.6%)	1 (6.2%)	>0.9999
SSB IgG	1 (0.8%)	4 (3.6%)	0.1917	1 (2.6%)	0 (0%)	>0.9999
a-MPO IgG	1 (0.8%)	0 (0%)	>0.9999	0 (0%)	0 (0%)	**-**
RNP IgG	0 (0%)	0 (0%)	**-**	0 (0%)	0 (0%)	**-**
dsDNA IgG	3/73 (4.1%)*	4/67 (5.6%)*	0.7097	0/25 (0%)*	0/13 (0%)*	**-**
AML IgG	3 (2.4%)	3 (2.7%)	>0.9999	2 (5.6%)	1 (6.2%)	>0.9999
MT IgG	1 (0.8%)	0 (0%)	>0.9999	0 (0%)	0 (0%)	**-**
ANCA IgG	3 (2.4%)	1 (0.9%)	0.6241	0 (0%)	1 (6.2%)	0.2963
CP IgG	2 (1.6%)	1 (0.9%)	>0.9999	0 (0%)	1 (6.2%)	0.2963
FAN IgG	2 (1.6%)	4 (3.6%)	0.4255	0 (0%)	1 (6.2%)	0.2963
LKM IgG	0 (0%)	0 (0%)	**-**	0 (0%)	0 (0%)	**-**

Autoantibodies were analyzed in individuals with LC (n = 237) and recovered (n = 54). COVID-19 severity was classified as mild/moderate or severe. Data are shown as number (n) and percentage (%). *Indicates missing data, resulting in fewer samples tested. p¹: comparison between mild/moderate and severe LC; p²: comparison between mild/moderate and severe recovered individuals.

### 3.3 Classification of COVID-19 severity

Nearly half of the LC group (47.3%) experienced severe COVID-19, compared with less than one-third of the recovered group (29.6%), reinforcing previous evidence that greater acute disease severity increases the likelihood of developing long COVID (**Table 1**). a-CL IgM was the most frequently detected autoantibody in severe cases, with significantly higher prevalence in recovered (31.2%) than in LC (9.8%; p = 0.0302) individuals. However, considering the other autoantibodies, the overall frequency of positivity was comparable between LC and recovered individuals, indicating that disease severity during the acute phase was not associated with increased autoantibody titers (**Table 2**). Nevertheless, no consistent severity-dependent pattern of autoantibody distribution was identified.

## 4 Discussion

Black and Hispanic individuals have been reported to be at higher risk of developing severe COVID-19 and are more likely to experience post-acute symptoms (17–20). However, no prior study has specifically examined the role of autoantibodies as biomarkers of LC in these populations. Our findings provide valuable insights into a population that remains underrepresented in studies investigating autoantibodies associated with long COVID. In our cohort, nearly one-quarter of participants identifying as Black or Mixed race exhibited detectable autoantibodies, a pattern that was consistently observed among those who had experienced severe COVID-19.

Similar observations, associating autoantibodies with disease severity but not with post-acute symptoms, have been reported in studies involving Caucasian cohorts from Europe and North America (10, 21–23). Collectively, these findings suggest that the profile of post-COVID-19 autoantibody expression may be consistent across ethnoracial groups, offering broader insights into the immunopathological mechanisms underlying post-infectious syndromes.

Autoantibodies have been extensively investigated as potential biomarkers of both acute COVID-19 and long COVID (LC). During the acute phase of SARS-CoV-2 infection, elevated levels of rheumatologic autoantibodies (e.g., rheumatoid factor, antinuclear antibodies) have been reported among hospitalized patients with severe COVID-19. Increased antiphospholipid and thyroid autoantibodies (e.g., anti-TPO) have also been described in association with disease severity (24). Moreover, neutralizing autoantibodies against type I interferons (IFN-α/ω) have been identified in approximately 10% of patients with life-threatening COVID-19, and their presence is linked to impaired antiviral responses and increased mortality (25, 26). Subsequent meta-analyses have confirmed a higher prevalence of these antibodies among patients with severe or critical disease (7–14%) compared with mild or moderate cases (27).

In the post-acute phase, however, large-scale studies that include healthy and recovered control groups, with varying degrees of acute disease severity and using diverse methodological approaches have not found consistent associations between autoantibodies and LC (5, 15, 16, 28–31). Although our cohort did not reveal a consistent association between autoantibodies and long COVID, recent studies indicate that LC patients may exhibit impaired humoral immunity following SARS-CoV-2 infection. Reduced anti-spike and neutralizing antibody responses, as well as lower IgG3 levels, have been reported in individuals with persistent symptoms compared with recovered controls (32, 33). These findings suggest that long COVID may involve insufficient antiviral immune mechanisms, underscoring the need for comprehensive T- and B-cell immunophenotyping to clarify its pathophysiology. Nonetheless, some reports have identified increased levels of autoantibodies against G-protein coupled receptors (GPCRs) in individuals experiencing post-acute neurological symptoms, persisting up to 12 months after infection (34, 35). These findings suggest that autoantibodies may be linked to specific symptom clusters rather than being universally associated with LC. Furthermore, when present, these autoantibodies do not necessarily imply a pathogenic role. For instance, persistent antiphospholipid antibodies in individuals with LC were not associated with thrombotic events in this and in previous studies (15, 29, 36). Collectively, these observations suggest that autoantibodies may serve as biomarkers of particular clinical phenotypes rather than indicators of LC pathogenesis itself.

The slightly lower prevalence of autoantibodies in our LC population (~24%) compared to previous reports (ranging from 30–60%) may be partially explained by methodological rather than ethnoracial differences (37, 38). In particular, we adopted more stringent cutoff values for positivity, following clinical criteria used for autoimmune syndromes (37, 38). For instance, in the case of a-CL and a-β2GP1 antibodies, we used the threshold defined for antiphospholipid syndrome (≥20 GPL/MPL), which is considerably higher than the manufacturer’s suggested reference value (e.g., 8 GPL/MPL). This conservative approach reduced the detection of low-titer or transient antibodies with uncertain clinical relevance. In addition, sample collection in our study was performed during the late post-acute phase, with a median of 1–3 months after symptom onset. At this stage, transient autoantibody responses may have already declined, particularly in individuals with self-limited immune activation (6, 28). In summary, both timing of sampling and cut-off range may have contributed to lower autoantibody detection. These findings collectively underscore the importance of standardized assay selection and harmonized cut-off values at the investigation biomarkers of LC.

We observed that a-CL IgM was the most frequently detected autoantibody across all study groups. Interestingly, the frequency was three times higher in recovered individuals (24%) compared to LC (8%), PPHC (8.1%) and historical healthy controls reported in previous studies (2–12%) (37, 38). Anticardiolipin antibodies (aCL), along with anti-β2 glycoprotein I (a-β2GP1) and lupus anticoagulant, are key markers of APS, an autoimmune condition characterized by recurrent thrombotic events and/or pregnancy morbidity (39). In individuals with a history of COVID-19, a-CL antibodies recognize different epitopes compared to classical APS (40), which may explain the lack of clinical implication. The presence of aCL antibodies may be a consequence of exposure of mitochondrial phospholipids during inflammatory responses induced by SARS-CoV-2 infection (10, 41–43). Cardiolipin can act as a neoantigen when translocated to the cell surface or released into the extracellular space. Such exposure is promoted by oxidative stress, apoptosis, and endothelial injury, pathophysiological processes frequently observed in COVID-19 (43, 44). These autoantibodies have also been reported in other viral infections, such as parvovirus B19, HCV, HIV and HBV, suggesting a non-specific mechanism of a-CL autoantibody production (45–47). Thus, the presence of these IgM autoantibodies may be a transient marker of acute cellular damage rather than autoimmunity triggered by SARS-CoV-2 (16, 48). The underlying cause of the increased aCL IgM frequency in recovered individuals remains unclear. Longitudinal studies investigating mitochondrial damage in recovered and LC participants will be essential to clarify these findings.

A major strength of our study is the inclusion of a large number of participants with well-matched pre-pandemic healthy individuals and recovered control groups, with similar symptom onset times. All cases were recruited during circulation of the alpha (B.1.1.7) and gamma (P.1) SARS-CoV-2 variants. However, our study has some limitations. First, expression of a-GPCRs, previously linked to neurological post covid symptoms, was not evaluated in our study. We acknowledge that the limited autoantibody panel and number of clinical clusters may have constrained our ability to detect the full spectrum of SARS-CoV-2 related autoantibody responses. Second, the questionnaire used in this study was developed before the release of the validated WHO instrument and constructed by a multidisciplinary team (49). Although the two tools differ in format, they are concordant in the most frequent symptoms and scales. Third, the present study employed the CDC definition of long COVID in effect during the recruitment period (symptoms persisting for more than four weeks). The CDC’s subsequent revision in 2024, extending the timeframe to three months or longer, underscores the ongoing efforts to standardize case definitions. Nonetheless, our supplementary analyses using the updated definition yielded comparable results, supporting the reliability of our conclusions regardless of definitional differences.

In summary, in Black/Mixed race participants, autoantibodies expression, yet associated with COVID-19 severity, seems not to be a biomarker of LC. The lack of association between these autoantibody profiles and clinical manifestations of long COVID indicates that, if present, the contribution of autoantibodies to symptom persistence is likely modest. Future work should prioritize longitudinal profiling to better delineate the dynamic interplay between autoantibodies, inflammatory markers, and clinical trajectories in this complex syndrome.

## Data Availability

The raw data supporting the conclusions of this article will be made available by the authors, without undue reservation.
