# Supplementary material for: Autoantibodies in long COVID in a black/mixed population compared with recovered and pre-pandemic controls
Source: Front Immunol. 2025 Dec 11;16:1684482. doi: 10.3389/fimmu.2025.1684482 (PMC12738873; doi:10.3389/fimmu.2025.1684482)
Supplement: Supplementary file 1 [file DataSheet1.pdf]

Supplementary Material

Table S1. Demographic and clinical characteristics of participants with long COVID (LC), recovered individuals, and pre-pandemic healthy controls (PPHC). LC patients included in this table exhibited symptoms persisting for three months or longer after the onset of SARS-CoV-2 infection. Abbreviations: LC, long COVID; PPHC, pre-pandemic healthy controls; BMI, body mass index; RT-PCR, reverse transcription polymerase chain reaction; Q1 and Q3, first and third quartiles. Values are expressed as n (%) or median (Q1, Q3).

	**COVID - 19**		
**Characteristic**	**LC**<br>N = 109	**Recovered**<br>N = 54	**PPHC**<br>N = 220
**Women (%)**	53 (49%)	21 (38.9%)	112 (51%)
**Age (median, IQR)**	54 (43, 66)	53 (40, 63)	40 (24, 54)
**Race**			
Mixed	60 (54.5%)	23 (42.6%)	75 (34.1%)
Black	35 (32%)	21 (38.9%)	57 (25.9%)
White	12 (11%)	8 (15%)	43 (19.5%)
Asian	2 (1.8%)	1 (1.9%)	-
Indigenous	-	1 (1.9%)	
Not declared	-	-	45 (20.5%)
**COVID-19 vaccine**			
Yes	15 (13.8%)	14 (25.9%)	-
No	94 (86.3%)	40 (74.1%)	-
**BMI**	28.8 (25.4, 33.4)	28.6 (24.6, 31.6)	-
**Months after disease onset** (median, IQR)	4.1 (3.4, 5.2)	3.0 (1.6, 4.5)	-
**Severity levels**			
Mild	36 (32.7%)	19 (35.2%)	-
Moderate	33 (30%)	19 (35.2%)	-
Severe	40 (37%)	16 (29.6%)	-
**COVID-19 tests**			
RT-PCR	94 (86%)	50 (94%)	-
Serology IgM/IgG	4 (3.7%)	0 (0%)	-
Rapid Antigen Test	6 (5.5%)	3 (6.3%)	-
Clinical-epidemiologic	5 (4.5%)	1 (1.9%)	-

Characteristic	COVID - 19 LC N = 109	COVID - 19 Recovered N = 54	PPHC N = 220
**Comorbidities**			-
Hypertension	53 (49%)	26 (48.1%)	-
DM	25 (23%)	13 (24.1%)	-
Cardiopathy	6 (5.5%)	4 (7.4%)	-
Psychiatric disorder	11 (10%)	4 (7.4%)	-
Asthma	6 (5.5%)	0 (0%)	-

Table S2 – Prevalence of autoantibody repertoire in individuals with long COVID (LC), recovered, and pre-pandemic healthy controls (PPHC). LC patients included here exhibited symptoms for three months or more after SARS-CoV-2 infection onset. The table summarizes the number of COVID-19 positive (COVID-19) and pre-pandemic subjects showing the presence of autoantibodies measured by ELISA (anti-β2-Glycoprotein, anti-Cardiolipin, anti-Sm, anti-Scl-70, anti-SSA, anti-SSB) and by immunofluorescence (anti-dsDNA, anti-AML, anti-MT, anti-ANCA, anti-Cardiolipin, anti-FAN, anti-LKM). Values are presented as n (%). Statistical analyses: Pearson's Chi-squared or Fisher's exact test; [1]p-value for comparison between all individuals with a history of COVID-19 (LC + Recovered) and PPHC; [2]p-value for comparison among all three groups (LC, Recovered, and PPHC). Sample sizes: [#]124 samples tested; [$]113 samples tested.

Autoantibodies	COVID - 19 Total N = 163	COVID - 19 LC N = 109	COVID - 19 Recovered N = 54	PPHC N = 220	p-value[1]	p-value[2]
**β2GP1 IgG**	0 (0%)	0 (0%)	0 (0%)	4 (3.2%)[#]	0.033	0.048
**β2GP1 IgM**	1 (0.6%)	0 (0%)	1 (1.8%)	9 (7.3%)[#]	0.002	0.006
**CL IgG**	3 (1.8%)	1 (0.9%)	2 (3.7%)	6 (4.8%)[#]	0.181	0.209
**CL IgM**	19 (11.7%)	6 (5.5%)	13 (24.1%)	10 (8.1%)[#]	0.317	<0.001
**SM IgG**	0 (0%)	0 (0%)	0 (0%)	0 (0%)	-	-
**SCL-70 IgG**	0 (0%)	0 (0%)	0 (0%)	0 (0%)	-	-
**SSA IgG**	5 (3.1%)	2 (1.8%)	3 (5.5%)	1 (0.5%)	0.087	0.023
**SSB IgG**	2 (1.3%)	1 (1%)	1 (1.8%)	0 (0%)	0.180	0.180
**a-MPO IgG**	1 (0.6%)	1 (1%)	0 (0%)	2 (0.9%)	>0.999	>0.999

**RNP IgG**	0 (0%)	0 (0%)	0 (0%)	0 (0%)	-	-
**dsDNA IgG**	2 (2.1%)	2 (3.4%)	0 (0%)	9 (4.1%)	0.097	0.373
**AML IgG**	5 (3.65%)	4 (3.6%)	3 (5.5%)	1 (0.5%)	0.048	0.012
**MT IgG**	0 (0%)	0 (0%)	0 (0%)	1 (0.5%)	>0.999	>0.999
**ANCA IgG**	5 (3.1%)	4 (3.7%)	1 (1.8%)	2 (1.8%)[$]	>0.999	0.702
**CP IgG**	2 (1.2%)	1 (0.9%)	1 (1.8%)	2 (0.9%)	0.703	0.783
**FAN IgG**	5 (3.1%)	4 (3.7%)	1 (1.8%)	9 (4.1%)	0.278	0.927
**LKM IgG**	0 (0%)	0 (0%)	0 (0%)	0 (0%)	-	-

Table S3. Frequency of autoantibodies in individuals with long COVID (LC), recovered, and pre-pandemic healthy controls (PPHC). LC patients included here exhibited symptoms for three months or more after SARS-CoV-2 infection onset. Autoantibodies were analyzed in individuals with mild/moderate COVID-19 (n = 107), severe COVID-19 (n = 56), and pre-pandemic controls (n = 220). No relevant differences were observed for most autoantibodies, except for a-β2GP1 IgM (p = 0.010). Values are presented as n (%). Statistical analysis: [1]Pearson's Chi-squared or Fisher's exact test comparing all three groups (Mild/Moderate, Severe, and PPHC). Sample sizes: [#]124 samples tested; [$]113 samples tested.

Autoantibodies	COVID-19 Mild/Moderate N = 107	COVID-19 Severe N = 56	PPHC N = 220	p-value[1]
**a-β2GP1 IgG**	0 (0%)	0 (0%)	4 (3.2%)[#]	0.108
**a-β2GP1 IgM**	1 (1%)	0 (0%)	9 (7.3%)[#]	0.011
**a-CL IgG**	3 (2.8%)	0 (0%)	6 (4.8%)[#]	0.268
**a-CL IgM**	10 (9.3%)	9 (16.1%)	10 (8.1%)[#]	0.242
**a-SM IgG**	0 (0%)	0 (0%)	0 (0%)	-
**a-SCL-70 IgG**	1 (1%)	0 (0%)	0 (0%)	0.425
**a-SSA IgG**	3 (2.8%)	2 (3.6%)	1 (0.5%)	0.066
**a-SSB IgG**	2 (1.9%)	0 (0%)	0 (0%)	0.098
**a-MPO IgG**	1 (1%)	0 (0%)	2 (0.9%)	>0.999
**a-RNP IgG**	0 (0%)	0 (0%)	0 (0%)	-
**a-dsDNA IgG**	1 (1.8%)	1 (2.9%)	9 (4.1%)	0.318
**a-AML IgG**	3 (2.8%)	2 (3.6%)	1 (0.5%)	0.066
**a-MT IgG**	0 (0%)	0 (0%)	1 (0.5%)	>0.999
**a-ANCA IgG**	3 (2.8%)	2 (3.6%)	2 (1.8%)[$]	0.701
**a-CP IgG**	1 (1%)	1 (1.8%)	2 (0.9%)	0.787
**a-FAN IgG**	1 (1%)	4 (7.1%)	9 (4.1%)	0.079
**a-LKM IgG**	0 (0%)	0 (0%)	0 (0%)	-

Table S4. Clinical manifestations reported by patients with long COVID (n = 237). The table presents the most frequently reported symptoms during follow-up, including fatigue, myalgia, memory impairment, and headache. Less frequent symptoms, such as dermatological and gastrointestinal complaints, were also observed. Values are presented as n (%).

Clinical manifestations	Long COVID (n = 237)
Dyspnea	154 (63.6%)
Fatigue	154 (63.%)
Chest pain	108 (44.6%)
Body aches	99 (40.9%)
Cough	93 (38.4%)
Headache	71 (29.3%)
Memory loss	58 (23.9%)
Insomnia	55 (22.7%)
Hair loss	46 (19%)
Limited movement	41 (16.9%)
Dizziness	39 (16.1%)
Anorexia	34 (14%)
Loss of appetite	30 (12.4%)
Loss of smell	27 (11.2%)
Expectoration	20 (8.3%)
Dysphonia	18 (7.4%)
Dysphagia	10 (4.1%)

Table S5. Prevalence of autoantibody repertoire in COVID-19, long COVID (LC), recovered, and pre-pandemic healthy controls (PPHC). LC patients included here exhibited symptoms for one month or more after SARS-CoV-2 infection onset. The table summarizes the number of COVID-19 positive (COVID-19) and pre-pandemic subjects showing the presence of autoantibodies measured by ELISA (anti-β2-Glycoprotein, anti-Cardiolipin, anti-Sm, anti-Scl-70, anti-SSA, anti-SSB) and by immunofluorescence (anti-dsDNA, anti-AML, anti-MT, anti-ANCA, anti-Cardiolipin, anti-FAN, anti-LKM). Values are presented as n (%). Statistical analyses: Pearson's Chi-squared or Fisher's exact test; [1]p-value for comparison between total individuals with a history of COVID-19 vs PPHC; [2]p-value for comparison among the LC, Recovered, and PPHC. Sample sizes: #124 samples tested; $113 samples tested.

Autoantibodies	COVID-19			PPHC	p-value[1]	p-value[2]
	Total N = 291	LC N = 237	Recovered N = 54	N = 220		
**β2GP1 IgG**	0 (0%)	0 (0%)	0 (0%)	4 (3.2%)#	0.007	0.015
**β2GP1 IgM**	3 (1.0%)	2 (0.8%)	1 (1.8%)	9 (7.3%)#	0.001	0.002
**CL IgG**	7 (2.4%)	5 (2.1%)	2 (3.7%)	6 (4.8%)#	0.192	0.280
**CL IgM**	32 (10.1%)	19 (8.0%)	13 (24.1%)	10 (8.1%)#	0.364	0.001
**SM IgG**	0 (0%)	0 (0%)	0 (0%)	0 (0%)	-	-
**SCL-70 IgG**	3 (1.0%)	3 (1.2%)	0 (0%)	0 (0%)	0.263	0.336
**SSA IgG**	9 (3.1%)	6 (2.5%)	3 (5.5%)	1 (0.5%)	0.048	0.032
**SSB IgG**	6 (2.1%)	5 (2.1%)	1 (1.8%)	0 (0%)	0.039	0.069
**a-MPO IgG**	1 (0.3%)	1 (0.4%)	0 (0%)	2 (0.9%)	0.580	0.721
**RNP IgG**	0 (0%)	0 (0%)	0 (0%)	0 (0%)	-	-
**dsDNA IgG**	7 (3.9%)	7/140 (5%)	0/38 (0%)	9 (4.1%)	0.936	0.475
**AML IgG**	9 (3.1%)	6 (2.5%)	3 (5.5%)	1 (0.5%)	0.048	0.032
**MT IgG**	1 (0.3%)	1 (0.4%)	0 (0%)	1 (0.5%)	>0.999	>0.999
**ANCA IgG**	5 (1.7%)	4 (1.6%)	1 (1.8%)	2 (1.8%)$	>0.999	>0.999
**CP IgG**	4 (1.4%)	3 (1.2%)	1 (1.8%)	2 (0.9%)	0.703	0.711
**FAN IgG**	7 (3.9%)	6 (2.5%)	1 (1.8%)	9 (4.1%)	0.2787	0.654
**LKM IgG**	0 (0%)	0 (0%)	0 (0%)	0 (0%)	-	-

Table S6. Odds ratio (OR), 95% confidence intervals (CI), and p-values for the association between autoantibodies and symptom clusters in long COVID patients.

Symptoms clusters	OR	95% CI	P value
**Cardiorespiratory**			
CL IgM	14.111	0.38 to 5.27	0.6083
dsDNA IgG	15.838	0.17 to 15.16	0.6899
SSA IgG	18.739	0.20 to 17.31	0.5798
AML IgG	12.786	0.14 to 11.79	0.8283
FAN IgG	13.848	0.15 to 12.59	0.7730
CL IgG	0.538	0.08 to 3.58	0.5211
SSB IgG	4.12E+10	0.00 to Inf	0.9890
**Dysphagia/anorexia**			
CL IgM	31.274	1.06 to 9.22	**0.0386***
dsDNA IgG	0.718	0.08 to 6.66	0.7708
SSA IgG	0.949	0.10 to 8.90	0.9633
AML IgG	21.886	0.37 to 13.03	0.3896
FAN IgG	3.40E-03	0.00 to Inf	0.9874
CL IgG	17.932	0.18 to 18.16	0.6210
SSB IgG	2.83E-03	0.00 to Inf	0.9886
**Systemic involvement**			
CL IgM	0.549	0.18 to 1.66	0.2886
dsDNA IgG	7.58E+10	0.00 to Inf	0.8203
SSA IgG	0.770	0.08 to 7.33	0.7518
AML IgG	0.256	0.05 to 1.45	0.1234
FAN IgG	12.717	0.14 to 11.76	0.8323
CL IgG	3.55E+10	0.00 to Inf	0.9882
SSB IgG	0.761	0.08 to 7.48	0.8148
**Neurologic**			
CL IgM	18.190	18.19	0.2542
dsDNA IgG	13.353	0.26 to 6.82	0.3133
SSA IgG	31.020	0.34 to 28.01	0.3327
AML IgG	0.456	0.08 to 2.71	0.3874
FAN IgG	17.852	0.31 to 10.29	0.5167
CL IgG	11.198	0.17 to 7.35	0.9062
SSB IgG	30.892	0.32 to 29.40	0.3265

Table S7. Frequency of autoantibodies in participants with mild/moderate COVID-19. Autoantibodies were measured in individuals with long COVID (n = 125) and in recovered participants (n = 38). Data are presented as counts (n) and percentages (%). *Indicates missing data, resulting in a reduced number of samples tested. Statistical comparisons were performed using Pearson's Chi-squared or Fisher's exact test.

	**Mild/Moderate**		
	**Long Covid**	**Recovered**	
**Autoantibodies**	N = 125	N = 38	**p-value**
**β2GP1 IgG**	0 (0%)	0 (0%)	-
**β2GP1 IgM**	0 (0%)	1 (2.6%)	0.2331
**CL IgG**	3 (2.4%)	2 (5.6%)	0.3312
**CL IgM**	8 (6.4%)	8 (21%)	0.0132
**SM IgG**	0 (0%)	0 (0%)	-
**SCL-70 IgG**	1 (0.8%)	0 (0%)	>0.9999
**SSA IgG**	3 (2.4%)	2 (5.6%)	0.3312
**SSB IgG**	1 (0.8%)	1 (2.6%)	0.4130
**a-MPO IgG**	1 (0.8%)	0 (0%)	>0.9999
**RNP IgG**	0 (0%)	0 (0%)	-
**dsDNA IgG**	3/73 (4.1%)*	0/25 (0%)*	0.5680
**AML IgG**	3 (2.4%)	2 (5.6%)	0.3312
**MT IgG**	1 (0.8%)	0 (0%)	>0.9999
**ANCA IgG**	3 (2.4%)	0 (0%)	>0.9999
**CP IgG**	2 (1.6%)	0 (0%)	>0.9999
**FAN IgG**	2 (1.6%)	0 (0%)	>0.9999
**LKM IgG**	0 (0%)	0 (0%)	-

Table S8. Frequency of autoantibodies in participants with severe COVID-19. Autoantibodies were measured in individuals with long COVID (n = 112) and in recovered participants (n = 16). Data are presented as counts (n) and percentages (%). *Indicates missing data, resulting in a reduced number of samples tested. Statistical comparisons were performed using Pearson's Chi-squared or Fisher's exact test.

	Severe		
	**Long Covid**	**Recovered**	
**Autoantibodies**	N = 112	N = 16	**p-value**
**β2GP1 IgG**	0 (0%)	0 (0%)	-
**β2GP1 IgM**	2 (1.7%)	0 (0%)	>0.9999
**CL IgG**	2 (1.7%)	0 (0%)	>0.9999
**CL IgM**	11 (9.8%)	5 (31.2%)	0.0302
**SM IgG**	0 (0%)	0 (0%)	-
**SCL-70 IgG**	2 (1.8%)	0 (0%)	>0.9999
**SSA IgG**	3 (2.7%)	1 (6.2%)	0.4178
**SSB IgG**	4 (3.6%)	0 (0%)	>0.9999
**a-MPO IgG**	0 (0%)	0 (0%)	-
**RNP IgG**	0 (0%)	0 (0%)	-
**dsDNA IgG**	4/67 (5.6%)*	0/13 (0%)*	>0.9999
**AML IgG**	3 (2.7%)	1 (6.2%)	0.4178
**MT IgG**	0 (0%)	0 (0%)	-
**ANCA IgG**	1 (0.9%)	1 (6.2%)	0.2352
**CP IgG**	1 (0.9%)	1 (6.2%)	0.2352
**FAN IgG**	4 (3.6%)	1 (6.2%)	0.4929
**LKM IgG**	0 (0%)	0 (0%)	-

Table S9. Raw values of autoantibody assays in individuals with long COVID (LC), recovered participants, and pre-pandemic healthy controls (PPHC). Data include ELISA results (international units per milliliter, IU/mL) and indirect immunofluorescence (IIF) findings, specifying the reagent used and corresponding antibody titers.

**Molecules analyzed/method**																	
	**Indirect ELISA**										**IIF**						
**Group**	**β2GP1 IgG**	**β2GP1 IgM**	**CL IgG**	**CL IgM**	**SM IgG**	**SCL-70 IgG**	**SSA IgG**	**SSB IgG**	**a-MPO IgG**	**RNP IgG**	**dsDNA IgG**	**AML IgG**	**MT IgG**	**ANCA IgG**	**CP IgG**	**FAN IgG**	**LKM IgG**
LC	1	2	2	3	1	1	5	<1	<1	1		N	N	N	N	N	N
LC	2	3	3	10	1	1	5	2	1	1	N	N	N	N	N	N	N
LC	1	2	2	3	1	1	3	1	1	1	N	N	N	N	N	N	N
LC	16	7	24	13	1	1	6	1	1	1	N	N	N	N	N	N	N
LC	5	1	6	4	1	1	3	1	1	1	N	N	N	N	N	N	N
LC	2	2	2	5	1	1	5	2	1	1	N	N	N	N	N	N	N
LC	2	3	4	6	1	1	7	2	1	1	N	N	N	N	N	N	N
LC	3	5	4	10	1	1	4	34	<1	1	N	N	N	N	N	N	N
LC	2	2	1	5	1	1	7	1	1	1	N	N	N	N	N	N	N
LC	2	1	3	3	1	1	9	2	1	1	N	N	N	N	N	N	N
LC	3	2	5	3	1	1	5	3	1	1	N	N	N	N	N	N	N
LC	5	4	8	14	1	1	6	4	1	1	N	N	N	N	N	N	N
LC	2	1	6	1	1	1	9	1	1	1	N	N	N	N	N	N	N
LC	1	1	2	2	1	1	4	1	1	1	N	N	N	N	N	N	N
LC	1	1	3	2	1	1	7	2	<1	1	N	N	N	N	N	N	N
LC	2	3	3	7	1	1	4	1	1	1	N	N	N	N	N	N	N
LC	2	3	3	6	1	1	4	6	1	1	N	N	N	N	N	N	N
LC	3	3	4	8	1	1	4	1	1	1	N	N	N	N	N	N	N
LC	4	3	4	5	1	1	2	1	1	1	N	N	N	N	N	N	N
LC	3	7	5	12	1	1	5	1	1	1	N	N	N	N	N	N	N
LC	3	8	4	27	1	1	8	4	1	1	N	N	N	N	N	N	N
LC	2	2	3	3	1	1	13	3	1	1	N	N	N	N	N	N	N
LC	2	1	2	2	1	1	31	14	1	1	N	N	N	N	N	N	N
LC	4	4	5	7	1	1	5	2	1	1	N	N	N	N	N	N	N
LC	2	2	3	4	1	1	7	2	1	1	N	N	N	N	N	N	N
LC	1	1	2	2	1	1	3	1	1	1	N	N	N	N	N	N	N
LC	2	2	2	6	1	1	3	1	1	1	N	N	N	N	N	N	N
LC	1	2	2	2	1	1	5	1	1	1	N	N	N	N	N	N	N
LC	18	2	52	2	1	1	6	2	1	1	N	N	N	N	N	N	N
LC	3	1	2	2	1	1	5	2	1	1	N	N	N	N	N	N	N
LC	2	2	3	5	1	1	6	2	1	1	N	N	N	N	N	N	N

LC	2	3	5	5	1	1	4	3	1	1	N	N	N	N	N	N	N
LC	2	6	3	24	1	1	5	1	1	1	N	N	N	N	N	N	N
LC	2	3	3	6	1	1	11	1	1	1	N	N	N	N	N	N	N
LC	2	<1	4	1	1	1	14	3	1	1	P	N	N	N	N	N	N
LC	1	1	2	2	1	1	5	<1	1	1		N	N	N	N	N	N
LC	1	2	2	3	1	1	4	<1	1	1		N	N	N	N	N	N
LC	2	4	3	7	1	1	3	1	1	1		N	N	N	N	N	N
LC	4	1	4	3	1	1	4	2	1	1		N	N	N	N	N	N
LC	1	3	3	4	1	1	12	1	2	1		N	N	N	N	N	N
LC	2	1	2	2	1	1	8	1	<1	1		N	N	N	N	N	N
LC	1	1	2	3	1	1	3	1	1	1		N	N	N	N	N	N
LC	2	4	1	4	1	1	4	1	1	1		N	N	N	N	N	N
LC	2	3	3	5	1	1	4	1	1	1		N	N	N	N	N	N
LC	3	2	4	4	1	1	3	1	1	1		N	N	N	N	N	N
LC	2	4	2	6	1	1	3	1	<1	1		N	N	N	N	N	N
LC	1	1	1	1	1	1	20	1	<1	1		N	N	N	N	N	N
LC	2	9	4	21	1	1	4	1	1	1		N	N	N	N	N	N
LC	2	2	1	5	1	1	7	2	<1	1		N	N	N	N	N	N
LC	2	5	3	17	1	1	3	1	1	1		N	N	N	N	N	N
LC	3	2	3	3	1	1	1	1	1	1		N	N	N	N	N	N
LC	2	7	4	24	1	1	6	1	1	1		N	N	N	N	N	N
LC	2	3	3	9	1	1	18	3	1	1		N	N	N	N	N	N
LC	2	1	2	2	1	1	5	1	1	1		N	N	N	N	N	N
LC	2	3	3	6	1	1	2	<1	1	1		N	N	N	N	N	N
LC	3	2	5	3	1	1	2	1	1	1		N	N	N	N	N	N
LC	2	1	3	3	1	1	9	1	1	1		N	N	N	N	N	N
LC	3	2	3	3	1	1	5	1	1	1		N	N	N	N	N	N
LC	2	4	3	15	1	1	3	2	1	1		N	N	N	N	N	N
LC	2	3	2	6	1	1	4	1	1	1		N	N	N	N	N	N
LC	7	4	15	14	2	1	17	5	7	2		P - 1/160	N	P - 1/140	N	N	N
LC	2	1	3	4	2	1	19	3	1	2	N	N	N	N	N	N	N
LC	2	5	2	9	2	1	17	3	2	2	N	N	N	N	N	N	N
LC	2	2	3	6	2	1	4	2	2	2	N	N	N	N	N	N	N
LC	2	4	2	4	2	1	4	2	1	2	N	N	N	N	N	N	N
LC	2	3	3	7	2	1	19	1	1	2	N	N	N	N	N	N	N
LC	2	3	2	5	2	1	4	2	1	2	N	N	N	N	N	N	N
LC	2	1	2	2	2	1	3	1	1	2	N	N	N	N	N	N	N
LC	2	3	2	7	2	1	14	3	1	2	N	N	N	N	N	N	N
LC	2	2	3	5	2	1	6	3	1	2		N	N	N	N	N	N
LC	2	1	2	1	2	1	19	<1	1	2		N	N	N	N	N	N

LC	2	3	2	6	3	1	7	2	<1	2	N	N	N	N	N	P - 1/80	N
LC	2	2	3	4	3	1	9	3	1	3		N	N	N	N	P - 1/80	N
LC	2	2	3	4	6	1	17	<1	1	6	N	N	N	N	N	N	N
LC	3	9	5	37	10	1	15	4	1	10		N	N	N	N	N	N
LC	3	4	5	16	<1	1	7	2	1	1		N	N	N	N	N	N
LC	1	1	1	2	<1	1	3	1	<1	1		N	N	N	N	N	N
LC	3	5	4	11	<1	1	2	1	<1	1		N	N	N	N	N	N
LC	2	1	2	2	<1	1	3	1	1	1		N	N	N	N	N	N
LC	1	2	2	2	<1	1	3	2	<1	1	N	N	N	N	N	P - 1/320	N
LC	4	7	4	15	1	2	2	3	<1	1		N	N	P - 1/40	N	N	N
LC	3	2	3	3	1	2	5	1	1	1	N	N	N	N	N	N	N
LC	2	1	2	1	1	2	4	1	1	1	N	N	N	N	N	N	N
LC	2	1	4	4	1	2	9	2	1	1	N	N	N	N	N	N	N
LC	9	1	11	2	1	2	7	2	1	1	N	N	N	N	N	N	N
LC	6	14	8	65	1	2	4	2	1	1	N	N	N	N	N	N	N
LC	2	2	11	3	1	2	3	1	1	1	N	N	N	N	N	N	N
LC	2	2	2	3	1	2	6	5	1	1	N	N	N	N	N	N	N
LC	2	2	2	5	1	2	6	2	2	1	N	N	N	N	N	N	N
LC	10	4	4	6	1	2	2	2	1	1		N	N	N	N	N	N
LC	16	5	18	9	1	2	2	2	1	1		N	N	N	N	N	N
LC	2	2	2	2	1	2	2	2	1	1		N	N	N	N	N	N
LC	3	1	3	3	1	2	3	1	1	1		N	N	N	N	N	N
LC	4	2	5	3	1	2	3	2	1	1		N	N	N	N	N	N
LC	3	4	5	6	1	2	2	1	1	1		N	N	N	N	N	N
LC	9	2	11	4	1	2	6	3	2	1		N	N	N	N	N	N
LC	1	2	2	2	1	2	3	2	1	1		N	N	N	N	N	N
LC	2	<1	3	1	1	2	4	8	1	1		P - 1/40	N	N	N	N	N
LC	3	5	4	14	2	2	3	1	1	2	N	N	N	N	N	N	N
LC	2	4	5	3	2	2	10	2	1	2	N	N	N	N	N	N	N
LC	1	1	2	4	2	2	10	2	1	2	N	N	N	N	N	N	N
LC	4	13	5	47	2	2	7	3	1	2	N	N	N	N	N	N	N
LC	2	2	2	4	2	2	7	1	1	2	N	N	N	N	N	N	N
LC	2	2	4	5	2	2	10	2	1	2	N	N	N	N	N	N	N
LC	2	2	4	5	2	2	8	1	1	2	N	N	N	N	N	N	N
LC	1	1	2	3	2	2	6	2	1	2	N	N	N	N	N	N	N
LC	4	8	4	17	2	2	10	2	1	2	N	N	N	N	N	N	N
LC	3	2	6	9	2	2	21	2	1	1		N	N	N	N	N	N
LC	4	2	5	7	2	2	8	2	1	2		N	N	N	N	N	N
LC	4	6	6	18	2	2	3	2	1	2		N	N	N	N	N	N
LC	6	6	7	11	2	2	6	3	1	2		N	N	N	N	N	N

LC	2	2	2	4	2	2	4	2	1	2		N	N	N	N	N	N
LC	2	3	2	5	2	2	3	2	1	2		N	N	N	N	N	N
LC	2	1	3	2	2	2	8	3	1	2		N	N	N	N	N	N
LC	4	1	20	3	2	2	9	2	2	2		N	N	N	N	N	N
LC	4	1	7	2	2	2	5	2	1	2	N	P - 1/80	N	N	N	N	N
LC	6	6	5	14	3	2	5	7	1	3	N	N	P - 1/80	N	N	N	N
LC	2	3	3	5	3	2	7	1	1	3	N	N	N	N	N	N	N
LC	4	2	7	8	3	2	8	2	1	3	N	N	N	N	N	N	N
LC	3	3	4	17	3	2	11	14	1	3		N	N	N	N	N	N
LC	8	6	8	19	1	3	6	5	2	1	N	N	N	N	N	N	N
LC	2	4	2	13	1	3	4	1	1	1		N	N	N	N	N	N
LC	4	2	5	8	2	3	9	3	4	2	N	N	N	N	N	N	N
LC	7	21	8	53	2	3	5	5	1	2	N	N	N	N	N	N	N
LC	2	1	4	4	2	3	4	38	1	2	N	N	N	N	N	N	N
LC	7	6	47	13	2	3	6	2	2	2	N	N	N	N	N	N	N
LC	2	4	5	8	2	3	60	33	1	1	N	N	N	N	N	N	N
LC	2	3	2	6	2	3	11	2	1	2	N	N	N	N	N	N	N
LC	14	10	23	21	2	3	8	3	2	2	N	N	N	N	N	N	N
LC	3	6	6	14	2	3	10	3	2	2	N	N	N	N	N	N	N
LC	2	2	8	3	2	3	4	2	1	2	N	N	N	N	N	N	N
LC	5	12	6	28	2	3	5	2	1	2	N	N	N	N	N	N	N
LC	3	1	4	6	2	3	10	7	1	2	N	N	N	N	N	N	N
LC	4	4	4	7	2	3	4	3	1	2		N	N	N	N	N	N
LC	6	4	4	9	2	3	5	2	1	2		N	N	N	N	N	N
LC	4	2	3	5	2	3	3	2	9	2		N	N	N	N	N	N
LC	3	4	4	4	2	3	4	2	1	2		N	N	N	N	N	N
LC	2	4	4	6	2	3	5	4	1	2		N	N	N	N	N	N
LC	4	4	4	6	2	3	4	3	1	2		N	N	N	N	N	N
LC	2	1	3	2	2	3	4	5	1	2		N	N	N	N	N	N
LC	3	3	7	6	2	3	3	12	1	2		N	N	N	N	N	N
LC	3	2	4	2	2	3	4	31	1	2		N	N	N	N	N	N
LC	3	5	5	9	3	3	9	2	1	3	N	N	N	N	N	N	N
LC	5	3	5	10	3	3	9	3	1	3	N	N	N	N	N	N	N
LC	11	4	17	10	3	3	8	8	1	3	N	N	N	N	N	N	N
LC	4	5	6	15	3	3	6	3	2	3	N	N	N	N	N	N	N
LC	3	4	5	10	3	3	7	3	2	3	N	N	N	N	N	N	N
LC	9	2	10	6	3	3	3	19	1	3		N	N	N	N	N	N
LC	8	10	12	18	3	3	6	2	1	3		N	N	N	N	N	N
LC	2	2	4	4	3	3	10	2	2	2		N	N	N	N	N	N
LC	5	5	7	17	4	3	4	3	16	4	P	N	N	P - 1/140	N	N	N

LC	1	3	2	4	4	3	4	4	1	4	N	N	N	N	N	N	N
LC	2	<1	3	1	6	3	7	4	2	6	N	N	N	N	N	N	N
LC	4	2	5	3	1	4	4	2	1	1		N	N	N	N	N	N
LC	6	5	8	11	1	4	2	1	1	1		N	N	N	N	N	N
LC	2	1	2	2	1	4	6	2	2	1		N	N	N	P - 1/320	N	N
LC	3	4	3	6	2	4	2	2	<1	2	N	N	N	N	N	N	N
LC	2	8	3	21	2	4	5	2	1	2	N	N	N	N	N	N	N
LC	3	4	7	8	2	4	15	5	2	2	N	N	N	N	N	N	N
LC	2	1	5	4	2	4	10	2	1	2	N	N	N	N	N	N	N
LC	2	3	3	7	2	4	12	3	1	2	N	N	N	N	N	N	N
LC	3	1	5	2	2	4	14	2	1	2	N	N	N	N	N	N	N
LC	3	5	4	8	2	4	10	3	1	2		N	N	N	N	N	N
LC	2	2	3	2	2	4	5	2	2	2		N	N	N	N	N	N
LC	3	3	13	3	2	4	2	2	1	2		N	N	N	N	N	N
LC	3	1	3	2	2	4	4	9	1	2		N	N	N	N	N	N
LC	11	3	7	3	2	4	6	3	1	2		N	N	N	N	N	N
LC	3	2	3	3	2	4	4	3	1	2		N	N	N	N	N	N
LC	2	2	6	3	2	4	8	5	1	2		N	N	N	N	N	N
LC	6	2	7	2	2	4	4	2	1	2		N	N	N	N	N	N
LC	3	2	7	3	2	4	3	4	1	2		N	N	N	N	N	N
LC	12	6	18	10	3	4	6	2	2	3	N	N	N	N	N	N	N
LC	2	2	4	3	3	4	11	4	1	3	N	N	N	N	N	N	N
LC	10	6	4	10	3	4	7	2	1	3	N	N	N	N	N	N	N
LC	2	3	2	9	3	4	4	1	1	3	N	N	N	N	N	N	N
LC	4	3	6	3	3	4	8	3	1	3	N	N	N	N	N	N	N
LC	7	45	12	109	3	4	11	6	1	3	N	N	N	N	N	N	N
LC	3	6	3	13	3	4	3	21	1	1		N	N	N	N	N	N
LC	2	3	3	4	3	4	6	2	1	3		N	N	N	N	N	N
LC	3	7	4	22	4	4	4	5	1	4	N	N	N	N	N	N	N
LC	5	4	8	7	4	4	14	4	1	4	N	N	N	N	N	N	N
LC	4	5	4	12	4	4	6	2	2	4	N	N	N	N	N	N	N
LC	2	4	3	12	4	4	4	2	1	4	P	N	N	N	N	N	N
LC	2	3	3	4	7	4	6	2	1	7	N	N	N	N	N	N	N
LC	3	5	3	7	1	5	4	1	1	1		N	N	N	N	N	N
LC	2	1	2	1	2	5	5	1	1	2	N	N	N	N	N	N	N
LC	6	3	6	20	2	5	10	5	2	2	N	N	N	N	N	N	N
LC	4	4	4	10	2	5	6	4	1	2	N	N	N	N	N	N	N
LC	3	4	8	12	2	5	9	4	2	2	P	N	N	N	N	N	N
LC	3	5	6	9	3	5	18	9	1	3	N	N	N	N	N	N	N
LC	4	3	5	5	3	5	18	4	2	3	N	N	N	N	N	N	N

LC	6	2	4	5	3	5	3	2	1	3		N	N	N	N	N	N
LC	6	2	17	3	3	5	14	2	1	3		N	N	N	N	N	N
LC	7	5	11	10	3	5	8	3	1	3	N	P - 1/80	N	N	N	N	N
LC	2	3	6	3	3	5	6	3	1	1		N	N	P - 1/40	N	P - 1/80	N
LC	4	6	6	24	6	5	37	2	2	7	N	N	N	N	N	N	N
LC	3	6	5	15	2	6	6	4	1	2	N	N	N	N	N	N	N
LC	10	4	10	8	2	6	46	38	3	2	N	N	N	N	N	N	N
LC	3	2	4	3	3	6	5	3	1	3	N	N	N	N	P - 1/160	N	N
LC	2	5	4	11	3	6	10	2	1	3	N	N	N	N	N	N	N
LC	11	8	11	19	3	6	3	3	1	3		N	N	N	N	N	N
LC	2	2	3	4	4	6	5	1	2	4	N	N	N	N	N	N	N
LC	4	4	4	3	4	6	12	6	1	2	P	N	N	N	N	N	N
LC	6	2	8	3	4	6	3	10	1	4		N	N	N	N	N	N
LC	1	1	2	2	5	6	18	<1	1	5	N	N	N	N	N	N	N
LC	2	3	2	6	1	7	6	3	1	1		N	N	N	N	N	N
LC	3	1	3	3	2	7	4	4	1	2	N	N	N	N	N	N	N
LC	2	3	3	5	2	7	13	7	1	2	N	N	N	N	N	N	N
LC	2	1	8	4	2	7	12	2	2	2	N	N	N	N	N	N	N
LC	3	13	13	28	3	7	11	2	1	3	N	N	N	N	N	N	N
LC	2	3	4	4	3	7	28	3	2	2	N	N	N	N	N	N	N
LC	3	5	4	10	4	7	2	1	1	4		N	N	N	N	N	N
LC	2	1	3	2	4	7	8	9	1	4		N	N	N	N	N	N
LC	1	7	5	17	5	7	3	3	1	3	P	N	N	N	N	N	N
LC	2	2	4	6	3	8	9	6	1	3	N	N	N	N	N	N	N
LC	5	5	6	7	4	8	11	2	1	4	N	N	N	N	N	N	N
LC	3	2	4	3	5	9	13	19	2	3	P	N	N	N	N	N	N
LC	2	2	4	4	1	10	4	4	1	1	N	N	N	N	N	N	N
LC	5	13	10	41	2	10	9	4	1	2	N	P - 1/80	N	N	N	N	N
LC	3	3	6	14	7	10	7	3	1	2		N	N	N	N	P - 1/160	N
LC	3	2	6	4	3	12	39	9	2	3	N	N	N	N	N	N	N
LC	3	1	6	2	7	12	10	3	1	6		N	N	N	N	N	N
LC	13	4	17	8	18	17	7	3	1	9	N	N	N	N	N	N	N
LC	3	9	6	26	5	18	6	10	2	5	N	N	N	N	N	N	N
LC	2	2	4	4	8	18	6	3	1	5	N	P - 1/40	N	N	N	N	N
LC	2	1	5	3	7	19	12	4	1	7	N	N	N	N	N	N	N
LC	3	2	4	7	2	25	7	4	1	1	N	N	N	N	N	N	N
LC	4	6	12	8	17	30	8	11	1	2	N	N	N	N	N	N	N
LC	3	7	4	15	14	34	3	3	1	7		N	N	N	P - 1/40	N	N
LC	3	2	3	5	3		14	1	1	3	N	N	N	N	N	N	N

LC	1	1	3	8	1	<1	9	1	2	1	N	N	N	N	N	N	N
LC	2	2	2	2	1	<1	10	2	1	1	N	N	N	N	N	N	N
LC	2	16	4	125	1	<1	4	1	<1	1		N	N	N	N	N	N
LC	1	1	1	1	1	<1	9	2	<1	1		N	N	N	N	N	N
LC	4	1	5	2	1	<1	4	1	1	1		N	N	N	N	N	N
LC	1	1	2	9	1	<1	5	4	<1	1	N	N	N	N	N	P - 1/80	N
LC	1	2	2	2	<1	<1	2	<1	<1	1		N	N	N	N	N	N
PPHC	0	5	3	7	1	1	0	1	1	1	N	N	N	N	N	P - 1/80	N
PPHC					1	2	1	6	1	3	N	N	N		N	P - 1/80	N
PPHC					0	0	0	1	0	0	N	N	N	N	N	P - 1/80	N
PPHC					1	3	6	6	3	4	N	N	N	N	N	P - 1/80	N
PPHC	0	2	1	7	1	0	1	1	1	0	N	N	N		N	P - 1/80	N
PPHC	4	2	9	3	2	2	4	4	5	2	N	N	N	N	N	P - 1/180	N
PPHC	1	2	4	5	1	2	15	1	2	2	N	N	N	N	N	P - 1/160	N
PPHC					1	1	7	3	1	0	N	N	N	N	N	P - 1/160	N
PPHC	2	6	6	21	0	1	2	1	2	1	N	N	N	N	N	P - 1/160	N
PPHC					1	2	3	3	3	2	N	N	N	N	N	N	N
PPHC	3	7	7	12	1	1	1	2	30	2	N	N	N	P - 1/40	N	N	N
PPHC					1	3	2	8	4	6	N	N	N	P - 1/40	N	N	N
PPHC	12	4	18	4	1	1	2	6	2	3	P	N	P - 1/40	N	N	N	N
PPHC					1	3	4	5	2	4	N	N	N	N	N	N	N
PPHC					1	1	3	2	2	2	N	N	N	N	N	N	N
PPHC					1	2	5	5	2	3	N	N	N	N	N	N	N
PPHC					0	1	7	3	1	1	N	N	N	N	N	N	N
PPHC	3	2	7	6	2	2	3	10	3	2	N	N	N	N	N	N	N
PPHC	1	9	4	11	1	1	1	0	1	2	N	N	N	N	N	N	N
PPHC	0	3	3	1	0	0	0	0	2	2	N	N	N	N	N	N	N
PPHC	0	7	4	3	1	1	1	2	1	2	N	N	N	N	N	N	N
PPHC	0	2	4	6	1	0	2	1	2	1	N	N	N	N	N	N	N
PPHC	2	10	4	17	9	1	8	3	2	4	N	N	N	N	N	N	N
PPHC					1	2	1	5	2	4	N	N	N	N	N	N	N
PPHC	0	1	3	1	0	1	1	3	1	2	N	N	N	N	N	N	N
PPHC	3	5	7	7	1	2	5	4	1	3	N	N	N	N	N	N	N
PPHC					0	0	3	3	1	1	N	N	N	N	N	N	N
PPHC	5	8	8	13	0	2	3	6	3	4	N	N	N	N	N	N	N
PPHC					0	0	7	0	1	1	N	N	N	N	N	N	N

PPHC	0	0	6	1	4	1	3	4	2	2	N	N	N	N	N	N	N
PPHC					1	0	3	2	1	2	N	N	N	N	N	N	N
PPHC					1	2	3	5	1	4	N	N	N	N	N	N	N
PPHC					1	0	1	2	1	1	N	N	N	N	N	N	N
PPHC					0	1	1	2	2	2	N	N	N	N	N	N	N
PPHC	24	58	21	2	0	1	3	4	2	2	N	N	N	N	N	N	N
PPHC	0	4	3	10	0	0	1	0	1	1	N	N	N	N	N	N	N
PPHC					0	0	2	1	8	0	N	N	N	N	N	N	N
PPHC	0	2	8	2	1	2	7	3	1	1	N	N	N	N	N	N	N
PPHC					1	1	2	1	1	0	N	N	N	N	N	N	N
PPHC					0	1	3	5	1	2	N	N	N	N	N	N	N
PPHC	1	13	9	22	1	3	7	3	3	3	N	N	N	N	N	N	N
PPHC	0	10	7	1	0	1	3	0	3	1	N	N	N	N	N	N	N
PPHC					0	2	2	7	1	6	N	N	N	N	N	N	N
PPHC	2	4	8	8	1	2	0	2	3	3	N	N	N	N	N	N	N
PPHC	2	4	5	7	1	1	1	1	3	2	N	N	N	N	N	N	N
PPHC	3	7	7	13	1	1	3	1	2	1	N	N	N	N	N	N	N
PPHC					0	0	1	1	1	1	N	N	N	N	N	N	N
PPHC	6	15	9	23	1	4	8	2	3	3	N	N	N	N	N	N	N
PPHC					1	3	10	12	4	6	N	N	N	N	N	N	N
PPHC	1	9	7	23	0	1	2	2	2	2	N	N	N	N	N	N	N
PPHC	0	3	8	4	9	2	5	2	3	3	N	N	N	N	N	N	N
PPHC	0	2	4	3	0	1	0	1	1	1	N	N	N	N	N	N	N
PPHC	1	2	5	2	1	2	1	2	3	2	N	N	N	N	N	N	N
PPHC	2	4	11	6	1	1	1	0	2	1	N	N	N	N	N	N	N
PPHC	2	1	5	1	0	2	5	2	2	3	N	N	N	N	N	N	N
PPHC	1	3	5	6	0	1	3	1	2	2	N	N	N	N	N	N	N
PPHC	1	3	6	6	0	1	2	4	2	2	N	N	N	N	N	N	N
PPHC	2	4	7	4	1	1	2	4	2	3	N	N	N	N	N	N	N
PPHC	0	0	4	0	0	1	2	1	1	1	N	N	N	N	N	N	N
PPHC	0	5	4	12	1	1	2	0	1	1	N	N	N	N	N	N	N
PPHC	3	3	6	7	7	1	6	2	5	3	N	N	N	N	N	N	N
PPHC	4	5	6	2	1	3	4	2	20	4	N	N	N	N	N	N	N
PPHC	3	6	6	7	1	1	4	2	3	2	N	N	N	N	N	N	N
PPHC	5	4	7	5	2	1	2	2	3	3	N	N	N	N	N	N	N
PPHC	2	3	6	4	1	1	2	1	2	2	N	N	N	N	N	N	N
PPHC	3	0	7	0	0	2	1	1	2	6	N	N	N	N	N	N	N
PPHC	2	7	4	3	0	1	2	1	1	1	N	N	N	N	N	N	N
PPHC					1	1	1	3	2	2	N	N	N	N	N	N	N
PPHC	0	4	6	3	1	1	2	5	2	5	N	N	N	N	N	N	N
PPHC	0	1	6	2	1	1	2	5	2	2	N	N	N	N	N	N	N

PPHC	14	165	24	3	1	1	3	6	3	2	N	N	N	N	N	N	N
PPHC	2	1	10	17	2	2	3	5	2	3	N	N	N	N	N	N	N
PPHC	1	2	5	2	0	1	3	3	3	2	N	N	N	N	N	N	N
PPHC	1	3	5	14	0	2	5	3	3	3	N	N	N	N	N	N	N
PPHC	1	18	5	6	1	3	2	10	3	3	N	N	N	N	N	N	N
PPHC					1	3	4	11	2	8	N	N	N	N	N	N	N
PPHC					0	0	1	2	1	1	N	N	N	N	N	N	N
PPHC	0	1	6	5	1	2	2	1	2	2	N	N	N	N	N	N	N
PPHC	0	0	5	0	1	2	2	1	2	2	N	N	N	N	N	N	N
PPHC	1	12	7	20	0	1	1	1	1	2	N	N	N	N	N	N	N
PPHC	0	4	5	2	0	1	2	1	2	2	N	N	N	N	N	N	N
PPHC	3	4	8	8	1	1	2	3	2	2	N	N	N	N	N	N	N
PPHC					1	1	2	2	1	2	N	N	N	N	N	N	N
PPHC					0	0	3	1	2	1	N	N	N	N	N	N	N
PPHC	2	21	10	43	1	2	2	2	3	4	N	N	N	N	N	N	N
PPHC	2	3	6	7	1	2	1	1	2	1	N	N	N	N	N	N	N
PPHC	0	3	4	6	1	2	2	4	3	8	N	N	N	N	N	N	N
PPHC	0	1	9	2	1	1	2	2	3	2	N	N	N	N	N	N	N
PPHC	1	13	7	34	1	2	1	1	4	3	N	N	N	N	N	N	N
PPHC	1	2	6	3	0	1	9	1	2	2	N	N	N	N	N	N	N
PPHC					1	1	4	3	1	2	N	N	N	N	N	N	N
PPHC	3	5	7	3	1	1	2	2	2	2	N	N	N	N	N	N	N
PPHC	2	3	6	4	1	2	9	2	3	2	N	N	N	N	N	N	N
PPHC	0	5	5	7	1	1	1	1	2	2	N	N	N	N	N	N	N
PPHC					1	1	3	2	1	2	N	N	N	N	N	N	N
PPHC					0	0	4	3	2	0	N	N	N	N	N	N	N
PPHC	0	1	4	5	0	1	2	0	2	1	N	N	N	N	N	N	N
PPHC	5	11	5	20	1	2	2	1	2	2	N	N	N	N	N	N	N
PPHC	1	44	4	6	1	2	2	2	2	3	N	N	N	N	N	N	N
PPHC	7	1	9	0	1	2	2	6	2	3	N	N	N	N	N	N	N
PPHC	0	0	4	0	1	0	3	0	2	0	N	N	N	N	N	N	N
PPHC					1	2	2	6	1	2	N	N	N	N	N	N	N
PPHC	2	2	5	3	0	2	4	2	2	2	N	N	N	N	N	N	N
PPHC					5	7	3	12	5	7	N	N	N	N	N	N	N
PPHC	0	3	6	2	1	1	2	1	2	2	N	N	N	N	N	N	N
PPHC	4	3	9	8	1	1	3	1	2	1	N	N	N	N	N	N	N
PPHC					0	0	0	1	0	0	N	N	N	N	N	N	N
PPHC					0	0	1	1	1	1	N	N	N	N	N	N	N
PPHC	2	5	6	3	1	3	4	2	3	3	N	N	N	N	N	N	N
PPHC	34	3	24	7	1	2	4	10	2	3	N	N	N	N	N	N	N
PPHC	4	3	6	6	1	2	2	1	2	2	N	N	N	N	N	N	N

PPHC					2	2	0	13	5	8	P	N	N	N	N	N	N
PPHC	1	6	6	16	1	1	3	1	3	2	P	N	N	N	N	N	N
PPHC	0	2	7	3	1	1	3	5	2	3	P	N	N	N	N	N	N
PPHC	9	27	13	44	2	3	6	2	7	4	P	N	N	N	N	N	N
PPHC					2	2	1	11	2	6	N	P - 1/40	N		N	N	N
PPHC					1	1	1	3	1	2	N	N	N		N	N	N
PPHC					1	1	3	6	2	2	N	N	N		N	N	N
PPHC					0	0	1	1	1	0	N	N	N		N	N	N
PPHC					0	2	10	6	2	4	N	N	N		N	N	N
PPHC					0	0	1	1	1	0	N	N	N		N	N	N
PPHC					1	1	3	5	2	4	N	N	N		N	N	N
PPHC					2	2	4	4	2	4	N	N	N		N	N	N
PPHC					1	2	4	7	2	2	N	N	N		N	N	N
PPHC					0	5	1	6	1	6	N	N	N		N	N	N
PPHC					1	1	2	5	2	3	N	N	N		N	N	N
PPHC					0	0	1	1	0	1	N	N	N		N	N	N
PPHC					2	2	1	4	3	9	N	N	N		N	N	N
PPHC					0	1	1	4	2	5	N	N	N		N	N	N
PPHC					0	1	1	4	2	3	N	N	N		N	N	N
PPHC					1	0	1	1	1	1	N	N	N		N	N	N
PPHC					1	0	0	2	0	2	N	N	N		N	N	N
PPHC					1	3	2	4	1	3	N	N	N		N	N	N
PPHC					0	1	4	10	1	1	N	N	N		N	N	N
PPHC					1	2	3	5	2	3	N	N	N		N	N	N
PPHC					0	1	3	5	2	4	N	N	N		N	N	N
PPHC					0	1	3	6	1	3	N	N	N		N	N	N
PPHC					1	3	3	8	2	5	N	N	N		N	N	N
PPHC					0	0	0	0	0	0	N	N	N		N	N	N
PPHC					1	2	2	5	2	4	N	N	N		N	N	N
PPHC	5	2	9	3	1	2	5	3	2	3	N	N	N		N	N	N
PPHC					2	2	3	8	2	4	N	N	N		N	N	N
PPHC	2	14	5	2	1	2	3	2	2	2	N	N	N		N	N	N
PPHC	3	17	7	15	1	1	2	2	3	2	N	N	N		N	N	N
PPHC	1	2	6	3	1	2	3	1	2	2	N	N	N		N	N	N
PPHC	1	2	6	4	1	1	3	2	2	2	N	N	N		N	N	N
PPHC	2	5	5	9	1	2	3	4	2	3	N	N	N		N	N	N
PPHC	6	0	12	0	1	3	4	3	2	3	N	N	N		N	N	N
PPHC	1	2	5	4	1	1	2	2	3	2	N	N	N		N	N	N
PPHC	3	8	6	3	1	5	6	2	1	4	N	N	N		N	N	N
PPHC	0	1	4	4	1	2	1	1	2	2	N	N	N		N	N	N
PPHC	6	5	10	8	0	1	0	0	1	1	N	N	N		N	N	N

PPHC					0	1	0	3	1	2	N	N	N		N	N	N
PPHC	2	4	6	9	1	2	4	3	3	2	N	N	N		N	N	N
PPHC	3	15	6	30	1	1	2	1	2	1	N	N	N		N	N	N
PPHC	2	2	6	6	0	2	4	2	3	2	N	N	N		N	N	N
PPHC	3	5	3	2	0	1	4	1	2	2	N	N	N		N	N	N
PPHC	0	1	4	2	0	1	3	5	2	2	N	N	N		N	N	N
PPHC	0	1	4	1	0	2	3	7	2	4	N	N	N		N	N	N
PPHC	1	21	5	19	0	1	3	0	2	1	N	N	N		N	N	N
PPHC					2	1	3	3	1	1	N	N	N		N	N	N
PPHC	10	10	8	0	1	2	2	1	2	2	N	N	N		N	N	N
PPHC					3	5	3	10	3	6	N	N	N		N	N	N
PPHC	5	0	2	0	1	1	2	1	2	2	N	N	N		N	N	N
PPHC	0	5	5	13	0	2	2	2	3	2	N	N	N		N	N	N
PPHC	0	3	1	4	0	0	0	0	0	0	N	N	N		N	N	N
PPHC	1	3	5	12	2	2	3	4	2	4	N	N	N		N	N	N
PPHC	0	1	8	2	0	1	1	2	1	2	N	N	N		N	N	N
PPHC	1	2	2	2	0	0	1	1	1	0	N	N	N		N	N	N
PPHC					1	1	3	5	2	4	N	N	N		N	N	N
PPHC					1	2	2	2	1	2	N	N	N		N	N	N
PPHC					0	0	1	1	1	2	N	N	N		N	N	N
PPHC	30	36	22	2	1	1	3	10	2	3	N	N	N		N	N	N
PPHC					0	1	3	2	1	3	N	N	N		N	N	N
PPHC	1	3	6	4	0	1	2	13	2	3	N	N	N		N	N	N
PPHC	0	2	0	0	0	1	3	1	2	1	N	N	N		N	N	N
PPHC					8	2	0	7	4	4	N	N	N		N	N	N
PPHC					1	1	2	3	1	3	N	N	N		N	N	N
PPHC	0	4	1	5	0	0	1	1	0	2	N	N	N		N	N	N
PPHC	0	2	3	2	2	1	1	2	1	3	N	N	N		N	N	N
PPHC	0	1	3	1	3	1	2	2	1	2	N	N	N		N	N	N
PPHC	0	8	3	4	0	0	1	1	1	1	N	N	N		N	N	N
PPHC	31	45	23	4	0	0	1	1	1	1	N	N	N		N	N	N
PPHC					1	2	1	11	2	5	N	N	N		N	N	N
PPHC					3	1	2	5	3	3	N	N	N		N	N	N
PPHC	1	12	2	4	0	0	1	1	1	2	N	N	N		N	N	N
PPHC					0	1	3	7	1	3	N	N	N		N	N	N
PPHC	0	3	3	6	0	1	1	0	1	1	N	N	N		N	N	N
PPHC					0	1	2	5	1	2	N	N	N		N	N	N
PPHC					0	1	1	2	1	2	N	N	N		N	N	N
PPHC	0	8	6	2	0	0	1	1	0	1	N	N	N		N	N	N
PPHC					1	2	2	7	2	4	N	N	N		N	N	N
PPHC					2	1	242	6	1	6	N	N	N		N	N	N

PPHC					1	0	1	1	1	1	N	N	N		N	N	N
PPHC					0	0	0	0	0	0	N	N	N		N	N	N
PPHC					0	0	0	1	0	2	N	N	N		N	N	N
PPHC	0	0	0	0	1	1	0	8	2	5	N	N	N		N	N	N
PPHC	0	5	2	20	0	0	1	0	2	1	N	N	N		N	N	N
PPHC					0	0	4	1	1	0	N	N	N		N	N	N
PPHC	0	6	3	6	0	1	3	1	1	1	N	N	N		N	N	N
PPHC					0	1	4	1	1	1	N	N	N		N	N	N
PPHC					0	0	1	1	1	0	N	N	N		N	N	N
PPHC					2	1	5	2	2	2	N	N	N		N	N	N
PPHC					1	1	4	5	2	3	N	N	N		N	N	N
PPHC					1	2	6	3	2	9	N	N	N		N	N	N
PPHC	0	2	2	3	0	1	1	2	0	1	N	N	N		N	N	N
PPHC	30	41	21	4	0	0	1	1	0	0	N	N	N		N	N	N
PPHC					1	1	1	3	2	7	N	N	N		N	N	N
PPHC					1	1	3	4	1	3	N	N	N		N	N	N
PPHC					0	1	1	1	1	1	N	N	N		N	N	N
PPHC					0	0	1	0	0	0	N	N	N		N	N	N
PPHC					1	1	1	2	1	2	N	N	N		N	N	N
PPHC					0	0	0	1	1	0	N	N	N		N	N	N
PPHC					1	0	3	1	0	0	N	N	N		N	N	N
PPHC					1	1	2	5	2	3	P	N	N		N	N	N
PPHC	5	6	9	5	1	4	4	2	5	3	P	N	N		N	N	N
PPHC	4	11	6	9	0	1	2	3	2	1	P	N	N		N	N	N
PPHC	0	2	6	5	1	1	2	3	5	2	P	N	N		N	N	N
PPHC					2	3	3	7	4	4	N	N	N		P - 1/160	N	N
PPHC	5	4	10	11	2	3	8	3	6	3	N	N	N		P - 1/40	N	N
Recovered	5	2	6	11	3	2	169	8	2	4	N	N	N	P - 1/160	N	P - 1/160	N
Recovered	6	6	9	18	9	7	5	3	3	9	N	N	N	N	N	N	N
Recovered	4	8	7	23	4	5	24	14	2	2	N	N	N	N	N	N	N
Recovered	16	17	23	99	<1	<1	4	2	1	1	N	N	N	N	N	N	N
Recovered	4	3	6	5	1	1	5	5	1	1	N	N	N	N	N	N	N
Recovered	13	5	13	17	1	2	5	5	1	1	N	N	N	N	N	N	N
Recovered	4	7	8	39	7	7	4	2	1	7	N	N	N	N	N	N	N
Recovered	2	2	3	6	1	2	7	2	1	1	N	N	N	N	N	N	N
Recovered	6	4	7	8	1	2	11	4	1	1	N	N	N	N	N	N	N
Recovered	4	10	6	73	1	2	7	6	1	1	N	N	N	N	N	N	N
Recovered	4	2	6	6	3	3	13	8	1	3	N	N	N	N	N	N	N
Recovered	3	1	3	4	2	2	13	3	1	1	N	N	N	N	N	N	N
Recovered	4	4	6	14	3	3	16	2	1	3	N	N	N	N	N	N	N

Recovered	3	7	6	56	3	4	4	3	1	3	N	N	N	N	N	N	N
Recovered	2	1	4	4	2	3	13	2	1	2	N	N	N	N	N	N	N
Recovered	8	6	7	18	4	4	30	5	1	1	N	N	N	N	N	N	N
Recovered	3	2	3	5	3	3	5	4	1	3	N	N	N	N	N	N	N
Recovered	4	3	4	6	3	3	9	5	1	3	N	N	N	N	N	N	N
Recovered	3	2	8	7	3	3	7	2	1	3	N	N	N	N	N	N	N
Recovered	3	2	5	8	1	1	9	5	1	2	N	N	N	N	N	N	N
Recovered	7	4	8	8	24	34	11	6	1	8	N	N	N	N	N	N	N
Recovered	9	16	14	86	3	2	5	4	1	3	N	N	N	N	N	N	N
Recovered	5	4	6	11	3	2	4	3	1	3	N	N	N	N	N	N	N
Recovered	3	1	3	3	4	3	6	7	1	4	N	N	N	N	N	N	N
Recovered	5	4	4	10	2	7	4	8	2	2	N	N	N	N	N	N	N
Recovered	3	1	4	7	1	1	4	1	1	1	N	N	N	N	N	N	N
Recovered	3	<1	3	3	1	2	7	5	1	1	N	N	N	N	N	N	N
Recovered	3	1	5	3	3	3	4	3	1	3	N	N	N	N	N	N	N
Recovered	5	5	6	13	2	3	8	8	2	2	N	N	N	N	N	N	N
Recovered	3	4	4	12	4	3	5	4	1	4	N	N	N	N	N	N	N
Recovered	3	1	4	3	4	4	13	3	1	4	N	N	N	N	N	N	N
Recovered	4	3	5	9	2	4	8	3	1	2	N	N	N	N	N	N	N
Recovered	3	4	4	9	7	7	13	3	1	7	N	N	N	N	N	N	N
Recovered	3	6	5	19	1	11	8	2	1	1	N	N	N	N	N	N	N
Recovered	9	10	16	27	2	2	14	3	1	2	N	N	N	N	N	N	N
Recovered	2	2	3	5	1	1	4	2	1	1		N	N	N	N	N	N
Recovered	2	1	4	3	2	2	3	1	1	2		N	N	N	N	N	N
Recovered	2	1	4	6	2	1	12	3	1	2		N	N	N	N	N	N
Recovered	6	8	9	21	6	6	3	3	1	6		N	N	N	N	N	N
Recovered	4	7	5	15	1	2	4	1	1	1		N	N	N	N	N	N
Recovered	3	2	3	6	3	4	4	6	1	3		N	N	N	N	N	N
Recovered	5	10	7	39	9	9	6	4	1	9		N	N	N	N	N	N
Recovered	8	6	9	24	2	3	6	3	2	2		N	N	N	N	N	N
Recovered	3	3	5	6	2	4	3	1	1	2		N	N	N	N	N	N
Recovered	5	4	4	22	3	2	6	2	1	3		N	N	N	N	N	N
Recovered	5	9	4	12	2	2	14	3	1	2		N	N	N	N	N	N
Recovered	4	9	5	22	3	1	9	2	1	3		N	N	N	N	N	N
Recovered	4	1	3	3	2	6	3	14	1	2		N	N	N	N	N	N
Recovered	2	2	2	4	8	2	6	1	1	8		N	N	N	N	N	N
Recovered	2	1	3	4	4	5	19	62	1	1		N	N	N	N	N	N
Recovered	8	21	23	83	5	6	14	4	1	5	N	P - 1/80	N	N	N	N	N
Recovered	7	4	11	9	3	3	3	1	1	3	N	P - 1/80	N	N	N	N	N
Recovered	5	1	5	3	3	7	33	4	2	2	N	P - 1/40	N	N	N	N	N

Recovered	4	5	4	12	3	4	13	4	1	3		N	N	N	P - 1/320	N	N
